# A Tailored Hydrogel With Local Glycemia Management, Antioxidant Activity, and Photothermal Antibacterial Properties for Diabetic Wound Healing

**DOI:** 10.1002/advs.202414161

**Published:** 2025-03-05

**Authors:** Bangguo Zhou, Yangying Duan, Wenhao Li, Tao Chen, Jincheng Wang, Manting Cao, Guohao Lin, Ke Yang, Zhangqi Lai, Wencheng Wu

**Affiliations:** ^1^ Department of Radiology The First Affiliated Hospital College of Medicine Zhejiang University Hangzhou Zhejiang 310003 P. R. China; ^2^ Central Laboratory and Department of Medical Ultrasound Sichuan Academy of Medical Sciences, Sichuan Provincial People's Hospital University of Electronic Science and Technology of China Chengdu Sichuan 610072 P. R. China; ^3^ The Third School of Clinical Medicine (School of Rehabilitation Medicine) Zhejiang Chinese Medical University Hangzhou 70571 P. R. China

**Keywords:** antioxidant, Au‐CeO_2_ dumbbells, diabetic wound healing, hydrogel dressing, local glucose management, photothermal antibacterial

## Abstract

The management of chronic diabetic wounds is a complex challenge requiring local glycemic regulation, modulation of inflammation levels, and prevention of bacterial infections. Therefore, a multifunctional wound dressing with antioxidant, local glycocontrol, and antibacterial properties is developed to promote diabetic wound healing. This dressing is constructed by co‐loading gold‐cerium oxide (AuCeO_2_) dumbbells and glucose oxidase (GOx) into a reactive oxygen species (ROS)‐sensitive hydrogel matrix (ACG gels). In this system, Au‐CeO_2_ dumbbells effectively eliminate ROS, protecting cells from oxidative stress‐induced damage while exhibiting significant near‐infrared photothermal antibacterial activity. Additionally, the controlled release of GOx decreases blood glucose levels in the wound microenvironment, alleviating oxidative stress and inhibiting bacterial growth proliferation, thereby expediting the healing process. ACG gels demonstrated excellent wound healing‐promoting properties in two in vivo wound models, providing a promising and effective platform for diabetic wound management.

## Graphical abstract

A multifunctional wound dressing (ACG gels) with antioxidant, local glycocontrol, and antibacterial properties to promote diabetic wound healing. Au‐CeO_2_ dumbbells effectively eliminate ROS, protecting cells from oxidative stress‐induced damage while exhibiting significant near‐infrared photothermal antibacterial activity. Additionally, the released GOx decreases blood glucose levels in the wound microenvironment, alleviating oxidative stress and inhibiting bacterial growth proliferation, thereby expediting the healing process.

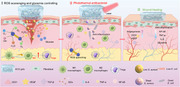

## 1 Introduction

Diabetes mellitus, distinguished by the presence of hyperglycemia, is a prominent disorder affecting the endocrine and metabolic systems and ranks among the top ten chronic diseases globally.^[^ ^1^, ^2^, ^3^ ^]^ Chronic diabetic wounds present a significant challenge to healthcare systems worldwide due to their high morbidity, mortality, and recurrence rates.^[^ ^4^ ^]^ Unlike other common wounds, chronic diabetic wounds are more vulnerable to bacterial infections and excessive accumulation of reactive oxygen species (ROS) due to their complex microenvironment, which impedes the healing process.^[^ ^5^ ^]^ For instance, local wound hyperglycemia can lead to elevated ROS levels, damaging normal cells and tissues and perpetuating inflammation.^[^ ^6^ ^]^ Thus, developing a multifunctional dressing that can regulate localized glycemic levels, provide efficient sterilization, and remove excess ROS to mitigate oxidative stress is crucial. Hydrogels have garnered considerable attention for their resemblance to soft tissue structures and have been engineered into various dressing forms, including 3D printed constructs, hydrogel films, and microneedles.^[^ ^7^, ^8^, ^9^ ^]^ Among these hydrogels, poly(vinyl alcohol) (PVA) is a physically crosslinked, one‐component hydrogel with a wide range of tunable mechanical, structural, and physical properties. PVA is widely used as a biomedical material due to its excellent biocompatibility, chemical stability, and high hydrophilicity.^[^ ^10^ ^]^ Furthermore, PVA hydrogels exhibit a slippery property akin to biological soft tissue and have been applied in various biomedical fields, including corneal implants,^[^ ^11^ ^]^ contact lenses,^[^ ^12^ ^]^ artificial meniscus,^[^ ^13^ ^]^ cartilage tissue replacement,^[^ ^14^ ^]^ and vitreous body replacement.^[^ ^15^ ^]^ However, the limited functional capacity of traditional hydrogels renders them inadequate to address the intricate and dynamic microenvironmental characteristics of diabetic wounds effectively. Hydrogel‐based composite systems have emerged as promising alternatives, integrating advanced functionalities, such as photothermal, photodynamic therapy, and gas‐based therapeutic approaches.^[^ ^16^, ^17^, ^18^, ^19^ ^]^

Excessive ROS, including hydrogen peroxide (H_2_O_2_), hydroxyl radicals (OH), and superoxide anions (O_2_^−^), induce oxidative stress, negatively affecting cellular viability and function, thereby impeding wound healing processes.^[^ ^20^, ^21^, ^22^ ^]^ Clinically, strategies for scavenging ROS using natural enzymes,^[^ ^23^ ^]^ vitamins,^[^ ^24^ ^]^ carotenoids,^[^ ^25^ ^]^ and phenolic compounds,^[^ ^26^ ^]^ have been extensively used to mitigate oxidative stress in treating inflammatory diseases. Despite their efficacy, these approaches have limitations, such as high production costs, sensitivity to environmental conditions, and limited catalytic stability.^[^ ^27^, ^28^ ^]^ To overcome these limitations, recent studies have demonstrated that various nanomaterials can function as artificial enzymes with biocatalytic and antioxidant properties. These materials include noble metals,^[^ ^29^ ^]^ metal‐organic frameworks,^[^ ^30^ ^]^ cerium (Ce),^[^ ^31^ ^]^ selenium,^[^ ^32^ ^]^ and polyphenol nanoparticles.^[^ ^33^ ^]^ Among these, cerium oxide nanoparticles (CeO_2_) nanoparticles (NPs) have attracted significant attention due to their mixed valence states.^[^ ^34^ ^]^ The abundant oxygen vacancies on the CeO_2_ surfaces facilitate the reversible transformation between Ce^3+^ and Ce^4+^, making it a promising ROS scavenger for treating diabetic wounds.^[^ ^35^ ^]^

Diabetic wounds are susceptible to bacterial infection due to the localized hyperglycemic microenvironment, which further delays wound healing.^[^ ^5^, ^36^ ^]^ While antibiotic therapy remains the cornerstone of treatment, its overuse poses risks such as immunosuppression and bacterial resistance.^[^ ^37^ ^]^ Recent research has focused on exploring nanomaterials with antibacterial properties, including copper nanoparticles,^[^ ^38^ ^]^ silver nanoparticles,^[^ ^39^ ^]^ zinc oxide nanoparticles,^[^ ^40^ ^]^ and nanographene oxide.^[^ ^41^ ^]^ Although metallic nanomaterials exhibit broad‐spectrum antibacterial properties, they pose significant toxicity risks to human tissues. Moreover, their antibacterial efficacy tends to decrease over time due to the gradual release of metal ions, complicating efforts to achieve sustainable treatment. Photothermal therapy has emerged as a promising alternative to conventional antibiotic therapy, offering minimal side effects, high controllability, and the potential to circumvent bacterial resistance.^[^ ^42^ ^]^ Photothermal agents can convert near‐infrared (NIR) light energy into thermal energy and effectively eradicating bacteria by disrupting cell membranes and denaturing proteins.^[^ ^43^ ^]^ Gold nanorods (Au NRs), a typical example of plasma‐based photothermal agents, have been extensively investigated for their ability to harvest broad‐spectrum light and facilitate effective energy conversion.^[^ ^44^ ^]^ Their high photothermal conversion efficiency and low toxicity make them ideal candidates for eradicating bacteria in diabetic wounds.

**Figure 1:**
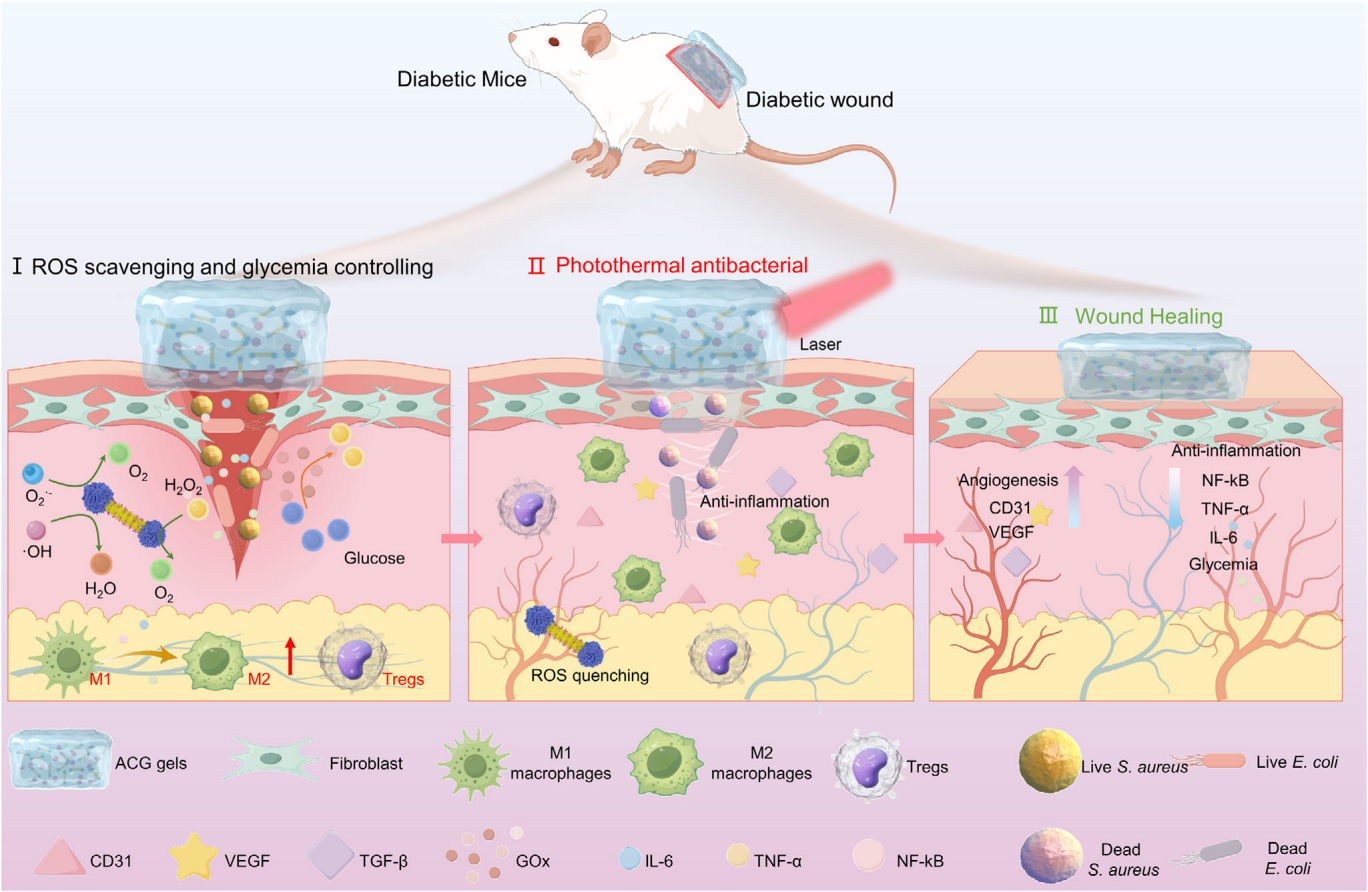
Schematic illustration of the mechanism by which ACG gels promote wound healing in infected diabetic wounds. Multifunctional ACG gels can I) rapidly remove ROS and regulate local blood glucose when applied to wounds, and II) exert excellent photothermal antimicrobial and anti‐inflammatory effects under NIR light, and III) accelerate wound healing.

Based on these considerations, a multifunctional hydrogel dressing was designed to accelerate the healing of diabetic wounds through localized hypoglycemia, ROS scavenging, and photothermal therapy (**Figure** **1**). To meet the requirements for antioxidant and photothermal therapies, we developed a novel nanoscale gold‐cerium oxide (Au‐CeO_2_) dumbbell structure. This design features gold nanorods with excellent photothermal conversion capabilities, while cerium oxide spheres positioned at each end exhibit superior antioxidant properties. Subsequently, Au‐CeO_2_ dumbbells and glucose oxidase (GOx) were co‐encapsulated within an ROS‐sensitive hydrogel matrix, resulting in the formation of a multifunctional wound dressing referred to as an ACG gel. When an ACG gel dressing is applied to the wound, it can rapidly remove excessive ROS and regulate the immune microenvironment by inducing the polarization of pro‐inflammatory M1‐type macrophages to anti‐inflammatory M2‐type macrophages. Additionally, they generate heat under NIR laser irradiation to kill bacteria such as *S. aureus* and *E. coli* thriving in wounds, preventing infections and facilitating wound healing. Moreover, locally released GOx metabolizes glucose within the wound environment to reduce glycemic levels, mitigate oxidative stress, and deprive bacteria of their energy sources to diminish the risk of infection, thus facilitating wound healing. Overall, the multifunctional hydrogel dressing presented in this study introduces an innovative platform for the treatment of diabetic wounds and demonstrates significant potential for future biomedical applications.

## 2 Results

### 2.1 Synthesis and Characterization of Au‐CeO_2_ Dumbbell and ACG Gel Dressings

**Figure 2:**
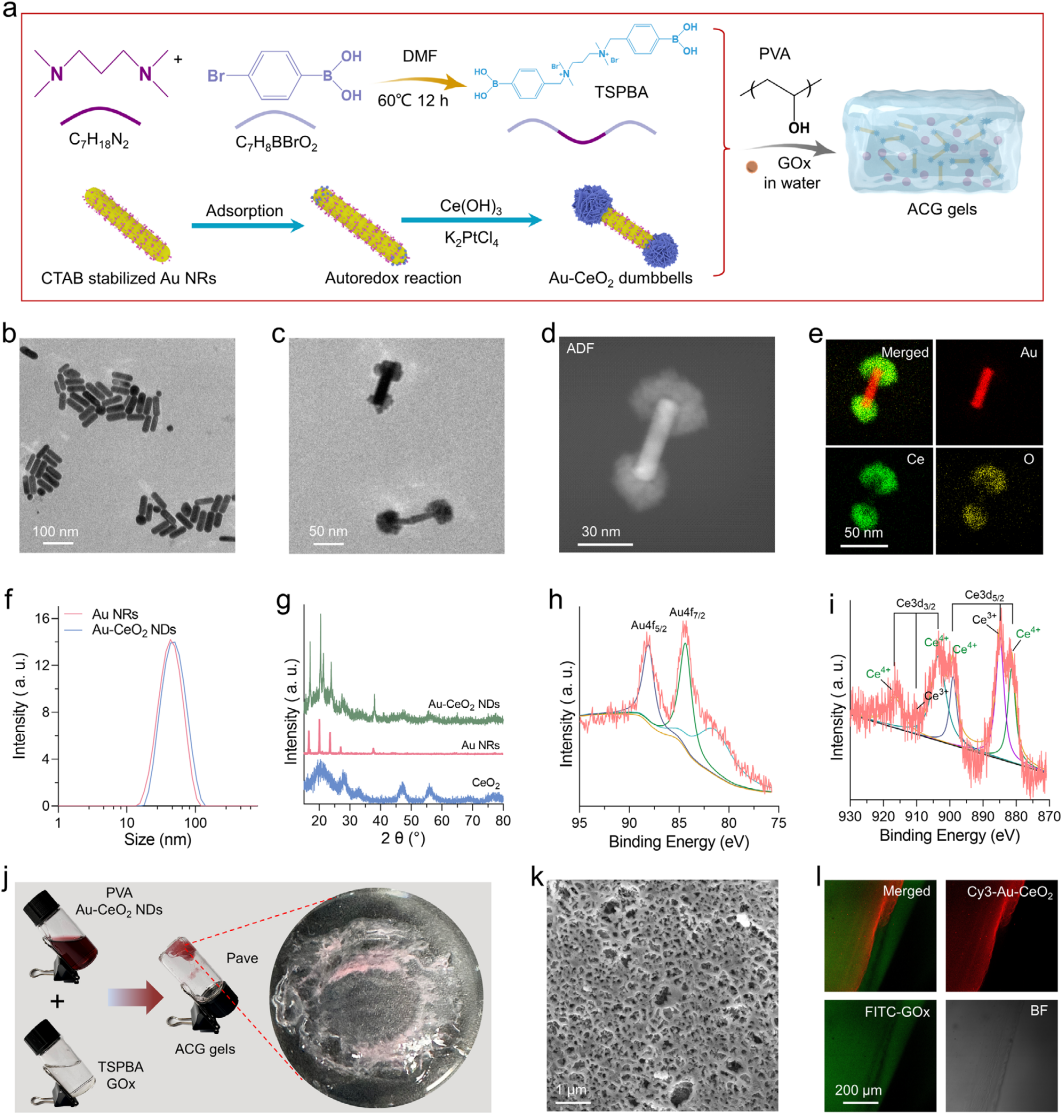
Preparation and characterizations of ACG gels. a) Schematic illustration of Au‐CeO_2_ dumbbells and ACG gel preparation. TEM image of b) Au nanorods and c) Au‐CeO_2_ dumbbells. d) High‐angle annular dark‐field image and e) corresponding mapping images of Au‐CeO_2_ dumbbells. f) DLS size distribution of different samples. g) XRD patterns of different samples. XPS analysis of h) Au and i) Ce. j) Digital photographs of different working fluids and the resulting gels after mixing. k) Cryo‐scanning electron microscopy (Cyro‐SEM) image of ACG gels. l) Representative CLSM fluorescence images of the ACG gels, with GOx labeled with fluorescein isothiocyanate (FITC) (green) and Au‐CeO_2_ labeled with Cy5.5 (red).

**Figure** **2**a illustrates the process for the selective coating of ceria onto a gold nanorod to produce the Au‐CeO_2_ dumbbell and the preparation of ACG gel dressings. The pregrown Au NRs were stabilized using a bilayer of cetyltrimethylammonium bromide (CTAB). Due to differences in crystal structure and curvature, the molecular chains of CTAB at the ends of the nanorods were less densely packed than those on the lateral surfaces, resulting in reduced steric hindrance and facilitating the access of other species to the Au NR ends. Consequently, PtCl_4_^2−^ ions preferentially adsorbed at the two ends of the Au NR. The ceria precursor (cerium acetate Ce(AC)_3_) underwent rapid hydrolysis to form Ce(OH)_3_ at temperatures exceeding 60 °C. This hydrolyzed product was subsequently oxidized by pre‐adsorbed PtCl_4_^2−^ through an auto‐redox reaction, enabling site‐selective nucleation and growth of CeO_2_. The interaction between PtCl_4_^2−^ and Ce(OH)_3_ ultimately yielded Au‐CeO_2_ dumbbell structure.^[^ ^45^ ^]^ The Au NRs used for ceria growth were ≈50 nm in diameter (Figure 2b). Transmission electron microscopy (TEM) images (Figure 2c) confirmed the formation of Au‐CeO_2_ dumbbell with a uniform morphology, showing selectively deposition of CeO_2_ on both ends of the Au NRs, thus exposing the side surface. To investigate the structural and chemical characteristics of the Au‐CeO_2_ dumbbell, high‐angle annular dark‐field scanning transmission electron microscopy (HAADF‐STEM) imaging and elemental mapping were performed. The HAADF‐STEM images and corresponding elemental mappings (Figure 2d,e) clearly demonstrate that each nanostructure comprised central Au NRs with Ce and O positioned at the extremities, as corroborated by the elemental profiles presented in Figure S1 (Supporting Information. Dynamic light scattering (DLS) analysis showed that both Au NRs and Au‐CeO_2_ dumbbells were well‐dispersed against aggregation in an aqueous solution, exhibiting narrow size distributions (Figure 2f). X‐ray diffraction analysis (Figure 2g) indicated that the CeO_2_ shell consisted of numerous small crystalline CeO_2_ nanoparticles. X‐ray photoelectron spectroscopy (XPS) analysis confirmed the presence of Au, Ce, and O (Figure S2, Supporting Information). High‐resolution XPS spectra of Au 4f (Figure 2h) revealed peaks at binding energies of 87.7 and 84.04 eV, corresponding to Au⁰. Similarly, high‐resolution Ce 3d spectra (Figure 2i) were deconvoluted into six peaks, four of which represented Ce⁴⁺ and two representing Ce^3^⁺, confirming the coexistence of mixed valence states with a predominance of Ce⁴⁺, favoring ROS scavenging’ to reduce wordiness. To fabricate the multi‐functional dressing, the synthesized Au‐CeO_2_ dumbbells and glucose oxidase (GOx) were co‐loaded into a ROS‐responsive degradable hydrogel scaffold (Figure 2j). This scaffold was synthesized by integrating cross‐linked poly(vinyl alcohol) (PVA) with ROS‐sensitive N1‐(4‐boronobenzyl)‐N3‐(4‐boronophenyl)‐N1, N1, N3, N3‐tetramethylpropane‐1,3‐diaminium (TSPBA). The TSPBA linkers hydrolyzed upon exposure to ROS, enabling controlled degradation. The rheological properties of the gel were evaluated, focusing on the elastic (G′) and viscous (G″) moduli. As anticipated, the G′ values increased significantly upon the introduction of TSPBA into the aqueous PVA solution, attributable to the formation of a network with the PVA chains (Figure S3, Supporting Information). The swelling rate of the ACG gel was 32.23%, indicating its effectiveness in absorbing wound exudates. Its adhesive strength, measured at 120 Pa through lap shear and burst pressure experiments, was sufficient for adhesion to skin wounds. The mechanical properties of the ACG gels were evaluated using tensile stress‐strain and rheological tests, revealing a tensile strength of ∼16.5 kPa, fracture strain of ∼220%, and elastic modulus of ∼400 Pa, making the gels well‐suited for dressings compatible with soft skin. The porous network microstructure of the bioactive scaffold was further corroborated by cryo‐SEM (Figure 2k). Additionally, fluorescence imaging of hydrogels containing FITC‐labeled GOx and Cy5.5‐labeled Au‐CeO_2_ dumbbells demonstrated that the piezoelectric catalysts and therapeutics were effectively encapsulated and uniformly distributed within the gels (Figure 2l).

### 2.2 ROS Scavenging and Modulation of Macrophages In Vitro

**Figure 3:**
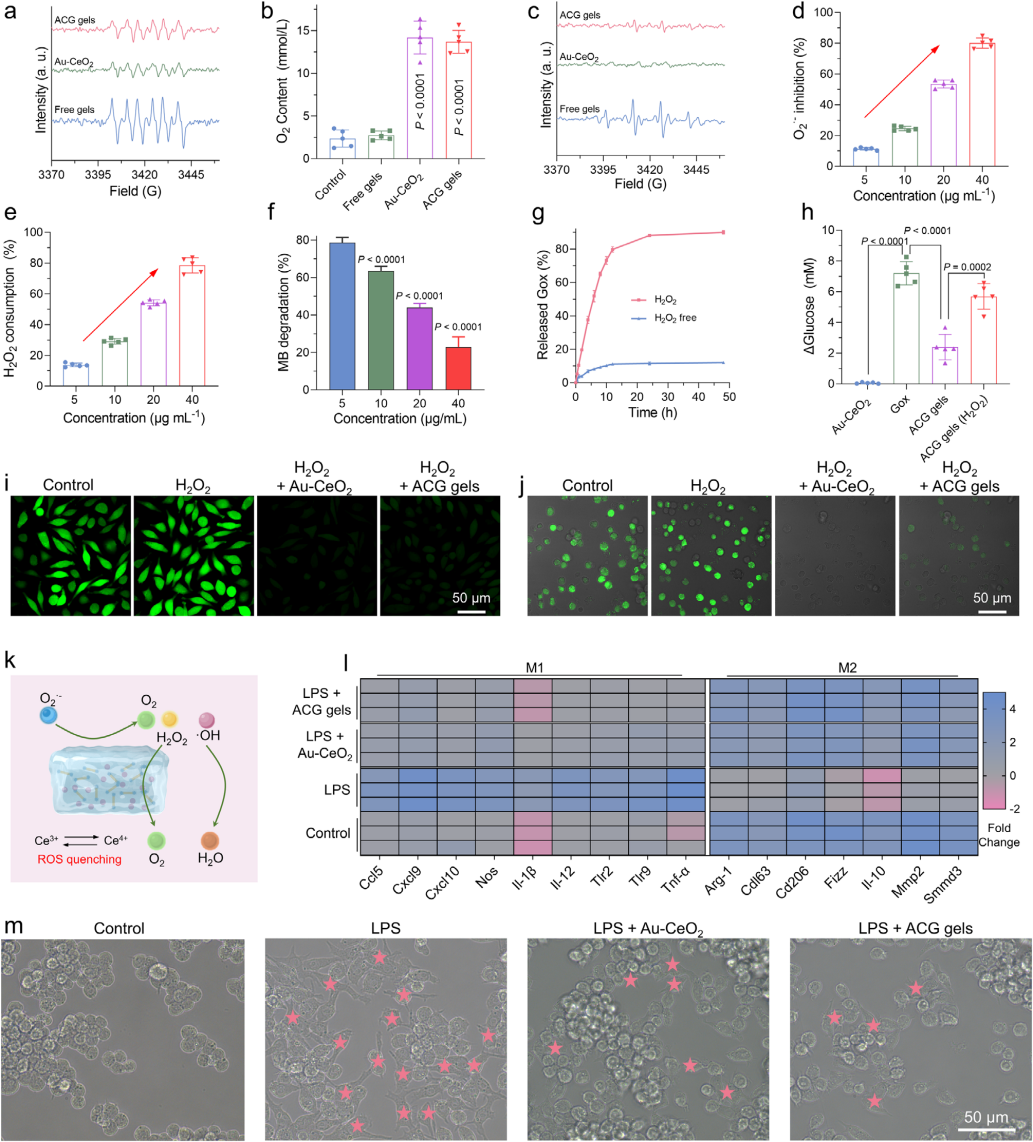
Performance evaluation of ACG gels in scavenging ROS and modulating macrophages. a) ESR spectra of O_2_^·−^ in different reaction systems. b) Oxygen generation capacity of different samples, as detected by an oxygen probe. c) ESR spectra of ·OH in different reaction systems. d) The O_2_^·‐^ scavenging ability of Au‐CeO_2_ dumbbells was evaluated using an O_2_^·−^‐specific total SOD assay kit with WST‐8. e) The H_2_O_2_‐scavenging ability of Au‐CeO_2_ dumbbells was measured by an H_2_O_2_‐specific total CAT assay kit. f) The efficiency of Fenton's reaction system for the degradation of methylene blue (MB) after the addition of different concentrations of Au‐CeO_2_ dumbbells. g) GOx release profiles of ACG gels in response to H_2_O_2_. h) Changes in glucose content in a standard glucose solution after the addition of different materials. Confocal fluorescent images of ROS levels in i) L929 and j) RAW264.7 cells with different treatments. Cells were stained with the ROS probe DCFH‐DA (green fluorescence) and DAPI (blue fluorescence). k) Schematic representation of ACG gels regulating M1‐type macrophage polarization towards M2‐type by scavenging ROS. l) The regulation of M1‐ and M2‐associated genes in LPS‐stimulated bone marrow (BM)‐derived macrophages (BMDMs) (M1 macrophages) after different treatments. m) The morphology of macrophages after being treated under different conditions. All data are presented as mean ± SD. Statistical significance was calculated by one‐way ANOVA. ns *P* > 0.05, ^*^ *P* < 0.05, ^**^ *P* < 0.01, ^***^ *P* < 0.001 and ****P < 0.0001.

The ROS scavenging capacity of various samples against superoxide anion (O_2_^−^), hydroxyl radical (·OH), and hydrogen peroxide (H_2_O_2_)‐commonly associated with inflammatory processes‐was initially assessed. As illustrated in **Figure** **3**a, both Au‐CeO_2_ dumbbells and ACG gels significantly reduced the specific O_2_^·−^ peak profile in the electron spin resonance (ESR) spectrum, indicating their effective capability to scavenge O_2_^·−^ by catalyzing its disproportionation into H_2_O_2_. The generated H₂O₂ was further eliminated through the catalase‐like (CAT‐like) activity of Au‐CeO_2_ dumbbells, as evidenced by the production of O₂ (Figure 3b). Additionally, ·OH was effectively scavenged through the redox cycling of Ce^3^⁺/Ce⁴⁺, as evidenced by the reduced signal intensity of ·OH in ESR spectra for reaction systems containing Au‐CeO_2_ dumbbells and ACG gels (Figure 3c). The concentration‐dependent ROS scavenging ability of Au‐CeO_2_ dumbbells was further confirmed through quantitative scavenging experiments for O_2_^·−^, ·OH, and H_2_O_2_ (Figure 3d–f). In the wound environment, excessive localized ROS (e.g., H_2_O_2_) triggered the release of GOx from the ACG gel, which consumed glucose and decreased the local blood glucose levels. After 48 h of incubation in a solution containing H_2_O_2_ (200 µM), the degradation rate of the hydrogel reached 94.36% (Figure S4, Supporting Information). Subsequently, the GOx release profiles of ACG gels were examined (Figure 3g). As expected, the release rate of GOx from the ACG gels increased following incubation with H_2_O_2_ solution, which was attributed to the ROS‐responsive degradation capability of the hydrogels. This result was indirectly supported by the enhanced glucose consumption capacity of ACG gels after the addition of H_2_O_2_ (Figure 3h). The ACG gel exhibited a higher glucose consumption capacity under slightly acidic conditions (Figure S5, Supporting Information), aligning with the slightly acidic environment of infected diabetic wounds and facilitating localized blood glucose regulation.

Following confirmation of the robust ROS‐scavenging capability of the ACG gels, their antioxidant potential in vitro was further assessed using L929 and BMDMs cell lines as models. Both Au‐CeO_2_ dumbbells and ACG gels effectively reduced ROS levels in H_2_O_2_‐pretreated L929 and BMDMs cells (Figure 3i,j), suggesting their potential for diabetic wound repair. Under inflammatory conditions, the Au‐CeO_2_ dumbbells and ACG gels significantly enhanced the viability of L929 cells and HUVEC (Figure S6, Supporting Information), confirming the cytoprotective properties of the Au‐CeO_2_ dumbbells. The elimination of ROS from the environment has been shown to facilitate the polarization of pro‐inflammatory M1 macrophages into anti‐inflammatory M2 macrophages, thereby mitigating the progression of inflammation (Figure 3k). RNA sequencing profiling results demonstrated that the treatment of lipopolysaccharide (LPS)‐stimulated M1 macrophages with Au‐CeO_2_ dumbbells or ACG gels revealed significant down‐regulation of M1‐associated genes (such as *Ccl5*, *Cxcl9*, *Cxcl10*, *Nos*, *Il‐1β*, *IL‐12*, *Tlr2*, *Tlr9*, and *Tnf‐)* (Figure 3l). Conversely, there was significant upregulation of M2‐associated genes, including *Arg1*, *Cd163*, *Cd206*, *Fizz*, *Il‐10*, *Mmp2*, and *Smad3* (Figure 3l). The expression of genes associated with macrophage polarization was confirmed by quantitative reverse transcription polymerase chain reaction (RT‐PCR) (Figure S7, Supporting Information). When M1 macrophages were treated with Au‐CeO_2_ dumbbells or ACG gels, the expression of M1 functional markers (TNF‐α and iNOS (inducible nitric oxide synthase)) was down‐regulated, but the expression of M2 functional markers (arginine‐1 (Arg‐1) and Fizz) was significantly up‐regulated. Intuitively, both the Au‐CeO_2_ dumbbells and ACG gels induced morphological changes in M1 macrophages, rendering them more akin to M2 macrophages (Figure 3m). Collectively, these findings suggest that the application of ACG gel dressings facilitates the transformation of M1 into M2 macrophages by scavenging excess ROS in diabetic wounds, thereby reshaping the inflamed immune milieu and promoting diabetic wound healing.

### 2.3 Photothermal and Antibacterial Properties

**Figure 4:**
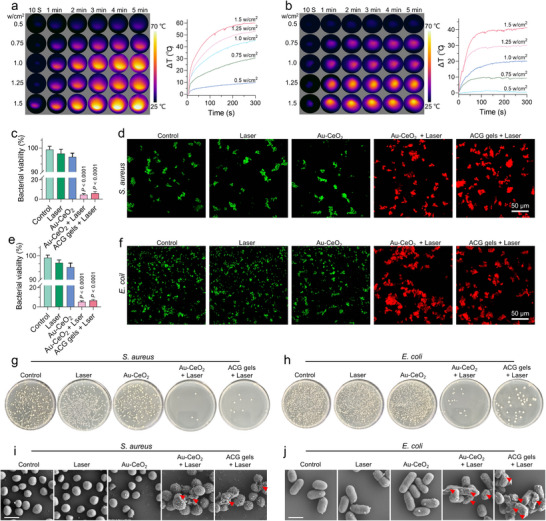
Evaluation of the photothermal antimicrobial performance of ACG gels. Real‐time infrared thermal images and corresponding photothermal curves of a) Au‐CeO_2_ dumbbells (200 ppm) and b) ACG gels (200 ppm) irradiated by a 1.5 W cm^−2^ NIR laser for 5 min. Quantification of the relative bacterial survival rate for c) *S. aureus* and d) *E. coli* after different treatments. Fluorescence microscopy images of e) *S. aureus* and f) *E. coli* stained with SYTO9/PI dyes after different treatments (all live bacteria stained green with SYTO9, and dead bacteria stained red with PI). Photographs of g) *S. aureus* and h) *E. coli* bacterial colonies after different treatments. SEM images of i) *S. aureus* and j) *E. coli* bacterial colonies after different treatments. The red arrow indicates damage to the structure of the bacterial cell membrane after photothermal therapy. Scale bar, 2 µm. All data are presented as mean ± SD. Statistical significance was calculated by one‐way ANOVA. ns *p* > 0.05, ^*^ *p* < 0.05, ^**^ *p* < 0.01, ^***^ *p* < 0.001, and ^****^ *p* < 0.0001.

Au NRs are widely used as photothermal agents. To evaluate the photothermal effect of the Au‐CeO_2_ dumbbells, an 808 nm NIR laser was used to irradiate a solution containing these dumbbells. At different laser power densities, the temperature of the Au‐CeO_2_ dumbbell solution (200 ppm) increased over five minutes of irradiation (Figure 4a,b). Even at relatively low laser power (1W cm^−2^), the solution temperature increased by ≈40 °C in five minutes, indicating the excellent photothermal conversion efficiency of Au‐CeO_2_ dumbbells. Benefiting from their special dumbbell structure, Au‐CeO_2_ showed superior photothermal heating properties compared to other typical photothermal agents such as Prussian blue and polydopamine nanoparticles (Figure S8, Supporting Information).

### 2.4 To kill the Bacteria in Wounds

Notably, the photothermal properties of the Au‐CeO_2_ dumbbells were retained even after being incorporated into hydrogels. The temperature of the ACG gels increased by 20 °C upon irradiation with a laser at a power density of 1 w cm^−2^ for 5 min, which is sufficient. The antibacterial properties of ACG gels were determined by direct contact with the bacterial suspensions. Staphylococcus aureus (*S. aureus*, gram‐positive) and Escherichia coli (*E. coli*, gram‐negative) were chosen as representative bacterial models. Antimicrobial activity was evaluated using the CCK‐8 assay, live/dead bacterial staining assay, and plate count methods. As illustrated in Figure 4c,d, NIR laser irradiation alone did not exhibit any antimicrobial effect on *S. aureus* or *E. coli*. Interestingly, the Au‐CeO_2_ dumbbells alone displayed a mild bactericidal effect on *S. aureus* and *E. coli* consistent with previous findings. Upon NIR laser irradiation, both the Au‐CeO_2_ and ACG gel groups exhibited significantly enhanced antimicrobial activity against *S. aureus* and *E. coli*. A similar trend was observed in the live/dead bacterial staining assays in the present study. In this assay, live bacteria with intact membranes emitted green fluorescence, whereas dead bacteria with damaged membranes emitted red fluorescence. Compared to the control group, the Au‐CeO_2_ and ACG gel groups exhibited a significantly higher percentage of red fluorescence, demonstrating that Au‐CeO_2_ and ACG gels can directly kill bacteria (Figure 4e,f). After NIR laser irradiation, the number of viable *S. aureus* and *E. coli* bacterial colonies decreased sharply in the Au‐CeO_2_ and ACG gel groups (Figure 4g,h). Furthermore, SEM images showed that the untreated *S. aureus* and *E. coli* cells had smooth and intact cell walls. After photothermal treatment, the images showed severe destruction of the bacterial structures (**Figure** **4**i,j), and most of the bacteria were killed by Au@CeO_2_ under NIR light irradiation, which is considered the main mechanism of photothermal antibacterial activity. The anti‐biofilm effect of the ACG hydrogel was also examined using crystal violet staining. Quantitative statistical analysis of biofilm biomass for *S. aureus* and *E. coli* under different treatments is shown in Figure S9 (Supporting Information). Compared to other control groups, the Au‐CeO_2_ + laser and ACG gel + laser treatments distinctly reduced the adhesion of both *S. aureus* and *E. coli* biofilms. The toxicity of photothermal treatment in L929 cells was also studied. The photothermal treatment resulted in significant toxicity in L929 cells (Figure S10, Supporting Information). Therefore, conventional photothermal therapy often damages normal cells surrounding wounds, causing inflammation. However, this issue can be mitigated by the unique antioxidant properties of Au‐CeO_2_ dumbbells. Moreover, the Au‐CeO_2_ dumbbells showed higher photothermal and antioxidant properties compared with Au nanorods and CeO_2_, respectively, suggesting that such separate structures of the Au‐CeO_2_ dumbbells could improve their catalytic performance and photothermal conversion efficiency (Figure S11, Supporting Information).

### 2.5 ACG Gels Decrease Oxidative Stress and Accelerate Diabetic Wound Healing

**Figure 5:**
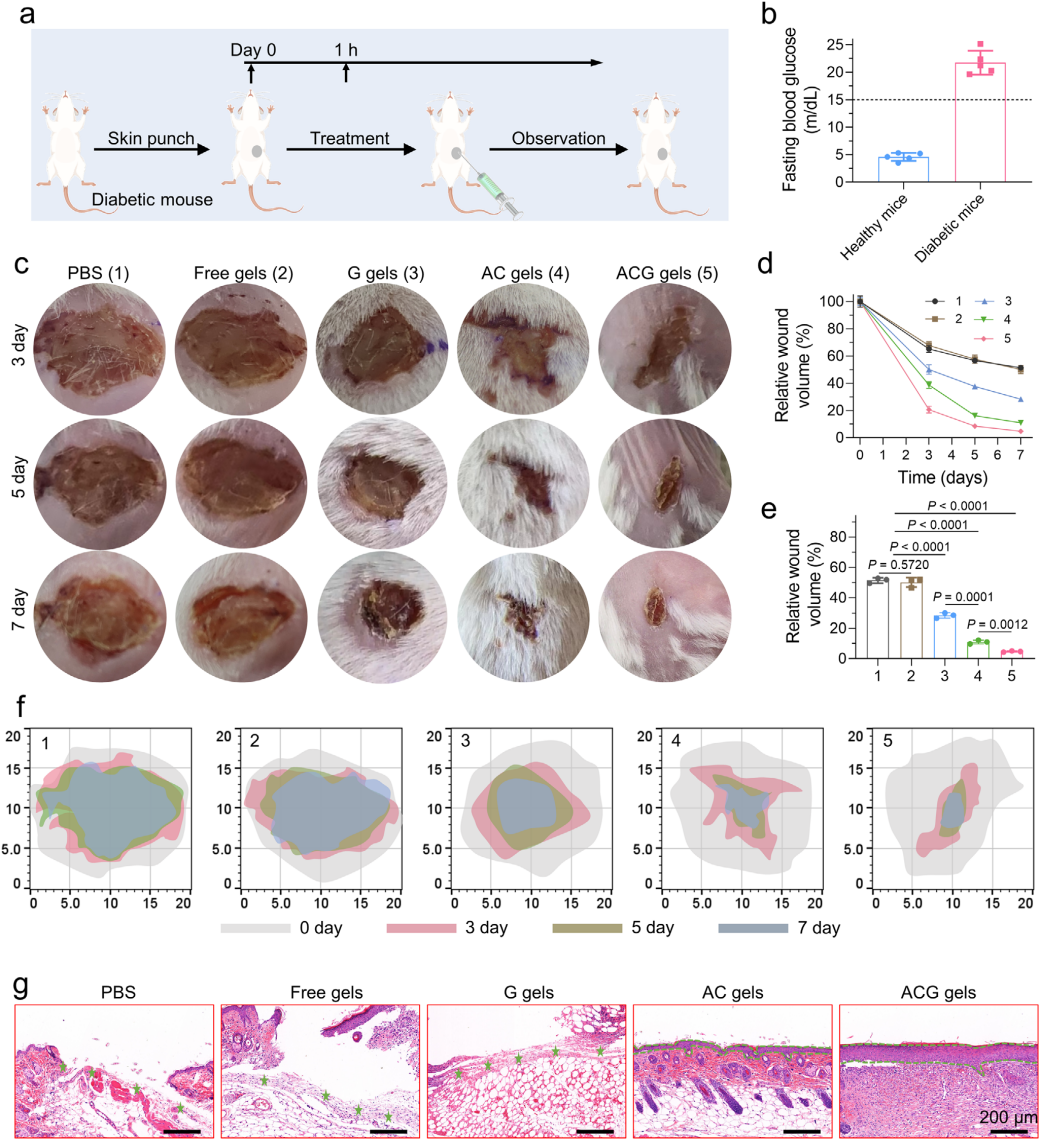
Performance evaluation of ACG gel for promoting diabetic wound healing via scavenging of ROS and local controlling glucose. (a) Schematic of the skin injury model construction and wound healing process promoted by ACG gels. (b) Fasting blood glucose levels of healthy and diabetic model mice (n = 5). (c) Representative digital images of diabetic wounds on days 3, 5, and 7 post‐wounding. (d) The wound boundary on days 3, 5, and 7 post‐wounding. (e) Wound healing percentages within 7 d post‐wounding in different groups. (f) Quantification of wound closure in diabetic mice on day 7 post‐wounding for all groups (n = 3). (g) H&E staining of wounds in different groups on day 3, with green stars and dotted lines indicating newly formed tissues. Data are presented as mean ± SD. Statistical significance was calculated by one‐way ANOVA. ns *P* > 0.05, *P < 0.05, **P < 0.01, ***P < 0.001 and ****P < 0.0001.

The microenvironment of diabetic wounds is characterized by elevated oxidative stress due to abnormal glucose metabolism. Mitigation of this oxidative stress can enhance wound healing. Motivated by the effective ROS‐scavenging properties of the Au‐CeO_2_ dumbbells observed in vitro, we explored the potential of an Au‐CeO_2_ dumbbell‐embedded hydrogel system (ACG gel) to accelerate the healing of diabetic wounds in vivo. Diabetic BALB/c mice with punched skin were randomly divided into four groups: control, free gel, G gels (GOx‐loaded gels), AC gels (Au‐CeO2 dumbbell‐loaded gels), and ACG gel (**Figure** **5**a). Fasting blood glucose levels in mice administered streptozotocin (STZ) at a dose of 50 mg k^−1^g daily for five consecutive days exceeded 15 mmol L^−1^ three weeks after the initial STZ injection, confirming the successful establishment of the diabetic mouse model (Figure 5b). Injectable hydrogels can be used to treat various irregular skin defects of different sizes, shapes, and depths. Upon injection into the wound site, these ACG gels demonstrated the ability to conform to the wound morphology and adhere effectively to the wound surface. Images of the wound site (Figure 5c,d) demonstrated that both G and AC gels significantly enhanced diabetic wound healing by mitigating oxidative stress. By day 7, the relative wound volumes in mice treated with G and AC gels were reduced to 28.3% and 10.9%, respectively, compared to the 50% relative wound volume observed in the control and free‐gel groups (Figure 5e,f). The commercially available conventional hydrogel (3m Tegaderm) exhibited similar pro‐healing effects as the designed free gels (Figure S12, Supporting Information). Notably, the relative wound volume of mice in the ACG gel group was further reduced to 4.7% by day 7, due to the synergistic wound healing‐promoting effect of scavenging ROS and localized reduced glycemia by the ACG gel. Histological analysis of neonatal wound tissue was conducted using hematoxylin and eosin (H&E) staining on the day following treatment. As shown in Figure 5g, the regenerated wound tissue in the ACG gel group exhibited the greatest thickness among all the experimental groups. Collectively, these findings corroborate the efficacy of ACG gels, which possess ROS‐scavenging and localized glycemia‐controlling properties, in enhancing wound healing.

**Figure 6:**
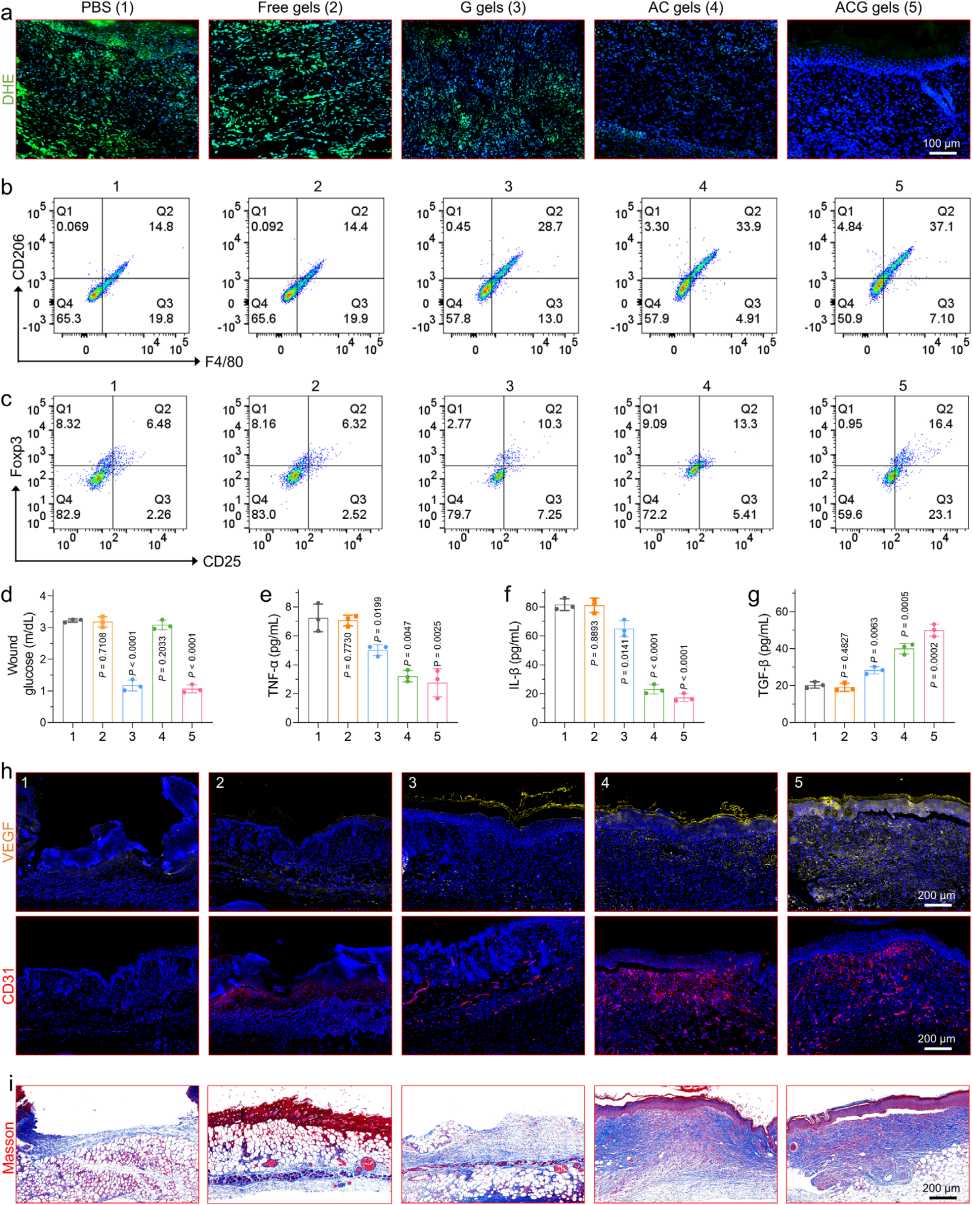
Mechanisms of ACG gel for promoting diabetic wound healing. a) Representative immunofluorescence images of DHE, VEGF, CD31, and Masson‐stained sections of skin tissues from mice at day 3 after different treatments. Representative flow cytometry data of b) M2‐type macrophages (F4/80^+^/CD206^+^ cells) and c) Tregs (CD25^+^/Foxp3^+^ cells gated on CD4^+^ cells) in wounds at day 3 after different treatments. d) Glucose content in wounds at day 3 after different treatments. Levels of e) TNF‐α, f) IL‐1β, and g) TGF‐β produced around the wound in different treatment groups at day 3 after different treatments. All data are presented as mean ± SD. Statistical significance was calculated by one‐way ANOVA. ns *P* > 0.05, ^*^ *p* < 0.05, ^**^ *p* < 0.01, ^***^ *p* < 0.001 and *p* < 0.0001.

Subsequently, a series of comprehensive studies were undertaken to elucidate the mechanism by which ACG gels facilitate wound healing. ROS levels at the wound site were assessed on postoperative days using dihydroethidium (DHE) staining. As expected, the diabetic wounds exhibited strong green fluorescence signals on day three post‐injury, signifying elevated ROS levels (**Figure** **6**a, top, and Figure S13, Supporting Information). The green fluorescence signals in diabetic wounds exhibited a marked reduction following treatment with G and AC gels, and this effect was further amplified by ACG gel treatment. In contrast, no such effect was observed in the free‐gel group. These findings suggest that ACG gels have superior ROS‐scavenging capabilities in vivo, facilitating sustained removal of excess ROS from diabetic wounds until complete wound healing is achieved. Next, a detailed investigation was conducted on in vivo alterations in the wound microenvironment following ACG gel dressing treatment. It is well established that M2 phenotype macrophages and regulatory T cells (Tregs) facilitate angiogenesis and collagen production, thereby playing crucial roles in tissue regeneration, including wound healing. Consequently, changes in the immune cell populations surrounding the wound were assessed on day 3 post‐treatment. Both M2 phenotype macrophages and Treg cells were most significantly increased in wounds treated with ACG gels (Figure 6b,c; Figure S14, Supporting Information). Meanwhile, the local blood glucose levels at the wound sites in mice treated with G and ACG gels were effectively downregulated compared to those in the other groups (Figure 6d), thereby contributing to a further reduction in oxidative stress. The ACG‐mediated localized blood glucose reduction was maintained for ≈48 h until the hydrogel was completely degraded (Figure S15, Supporting Information). The pro‐inflammatory cytokines, including tumor necrosis factor (TNF‐α) and interleukin‐1β (IL‐1β) were significantly downregulated in the ACG gels group, while the anti‐inflammatory cytokines (interleukin‐10, IL‐10) was obviously upregulated (Figure 6e–g). Improvement of the immune microenvironment significantly boosts neovascularization at the wound site, thereby promoting wound healing. As shown in Figure 6h and Figure S16 (Supporting Information), the expression of CD31 and VEGF was significantly elevated in the wounds treated with ACG gels compared to that in the other three groups, indicating enhanced neovascularization. The extent of collagen deposition within granulation tissue serves as a pivotal marker for wound healing. Figure 6i illustrates the results of Masson's trichrome staining, demonstrating a more pronounced and intense blue coloration indicative of collagen deposition in the ACG gel group than in the other groups. Quantitative analysis of the data (Figure S17, Supporting Information) revealed that the collagen deposition ratio was significantly higher in the ACG gel group (86.36%) compared to that in the G (36.04%) and AC gel groups (54.64%). These findings suggest that ACG gels can effectively improve the inflammatory immune microenvironment by locally regulating glycemic levels and scavenging ROS, thereby facilitating angiogenesis and collagen deposition and ultimately expediting the wound healing process.

### 2.6 ACG gels Promote Infected Diabetic Wound Healing via Photothermal Sterilization

**Figure 7:**
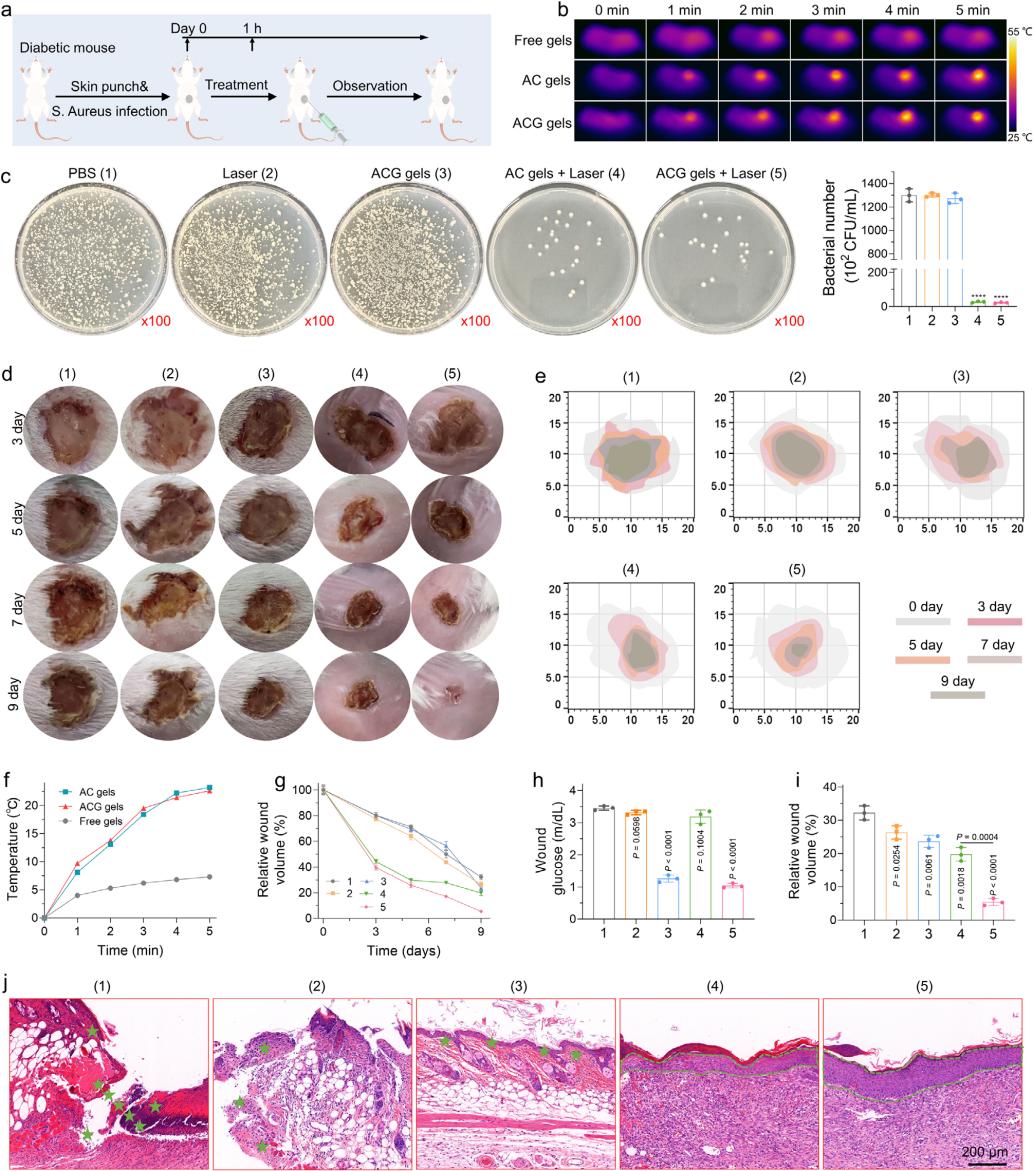
Performance evaluation of ACG gels for promoting diabetic wound healing under *S. aureus* infection. a) Schematic of skin injury model construction and wound healing process promoted by ACG gels. b) Real‐time infrared thermal images and e) thermal curves of infected wounds treated with free gels, AC gels, or ACG gels after 808 nm laser irradiation for 5 min (1.5 W cm^−2^). c) Photographs of bacterial colonies and corresponding quantification of bacterial numbers in the region of infection in different groups on day 3. d) Representative digital images of infected wounds on day 3, 5, 7, and 9 after different treatments. (1) PBS, (2) Laser, (3) ACG gels, (4) AC gels + Laser, and (5) ACG gels + Laser. f) Wound boundary observations on day 3, 5, 7, and 9 after different treatments. g) Monitoring of wound closure during the 9 days of each treatment. h) Quantification of infected wound closure in diabetic mice under infection at day 9 post‐wounding for all groups. i) Glucose content in wounds at day 3 after different treatments. j) H&E staining of the wounds in different groups on day 3, with green stars and dotted lines marking areas of newborn tissue. All data are presented as mean ± SD. Statistical significance was calculated using one‐way ANOVA. ns *p* > 0.05, ^*^ *p* < 0.05, ^**^ *p* < 0.01, ^***^ *p* < 0.001 and *p* < 0.0001.

Diabetic wounds are susceptible to bacterial infections during healing, with *S. aureus* being the most prevalent pathogen. Given the excellent in vitro photothermal antimicrobial capacity of the ACG gels, we investigated their therapeutic effectiveness using an *S. aureus*‐infected wound model. The model establishment and therapeutic processes are depicted in **Figure** **7**a. Preliminary experiments were conducted to assess the in vivo photothermal effects of various treatment modalities using thermal imaging, as shown in Figure 7b. In mice treated with free gels, the temperature at the wound site increased by ≈5 °C under 808 nm NIR laser irradiation for 5 min. Conversely, in groups treated with AC gels and ACG gels, the temperature at the wound site rose by 25 °C during the same time frame, demonstrating the superior in vivo photothermal properties of the Au‐CeO_2_‐based hydrogel (Figure 7f). Subsequently, the infected mice were randomly assigned to five distinct groups: (1) PBS, (2) laser, (3) ACG gel, (4) AC gel + laser, and (5) ACG gel + laser. Three days after treatment, all wounds exhibited a biofilm layer or a mixture of hydrogel and exudate. To assess bacterial viability in infected wounds post‐treatment, biofilms or mixtures were collected on day 3, and bacterial enumeration was performed using the spread plate method (Figure 7c). The bacterial colony counts in groups 4 and 5 were significantly lower than those in other groups, suggesting that effective photothermal sterilization was achieved by AC gel + laser or ACG gel + laser treatment. Alterations in the wound area were monitored to assess the therapeutic efficacy of different treatments in promoting infected wound healing (Figure 7d,e). Quantitative analysis of the wound area revealed a significant reduction in the diabetic wound area of mice in Groups 4 and 5 compared to those of the mice in the other groups on day 3 (Figure 7g). The commercially available 3 m Tegaderm hydrogel showed similar pro‐healing effects as designed free gels (Figure S18, Supporting Information). ACG gel treatment alone did not significantly accelerate the healing of infected diabetic wounds compared to the control and laser groups, suggesting that bacterial infection severely limits the therapeutic effect of ACG gels. Although a single photothermal treatment (AC gel + laser) rapidly inhibited bacterial infectious inflammation and promoted early‐stage wound healing, the rate of wound healing declined over time due to the localized high glycemic environment in diabetic wounds. By introducing GOx to reduce local wound glycemia, ACG gels demonstrated significantly enhanced therapeutic effects under laser irradiation, highlighting the importance of local glycemic control in diabetic wounds (Figure 7h). By day 9, the wound area in the AC gel + laser group reduced to 19.7%, whereas the wound epithelium in the ACG gel + laser group was completely covered and almost completely healed (Figure 7i). In addition, the thickness of the regenerated wound tissue in the ACG gel group was the greatest among the experimental groups, as observed in the histological analysis of H&E‐stained neonatal wounds (Figure 7j). Collectively, ACG gels effectively kill bacteria, inhibit inflammation, and locally regulate glycemia with the assistance of NIR irradiation, thereby accelerating healing in diabetic wounds.

**Figure 8:**
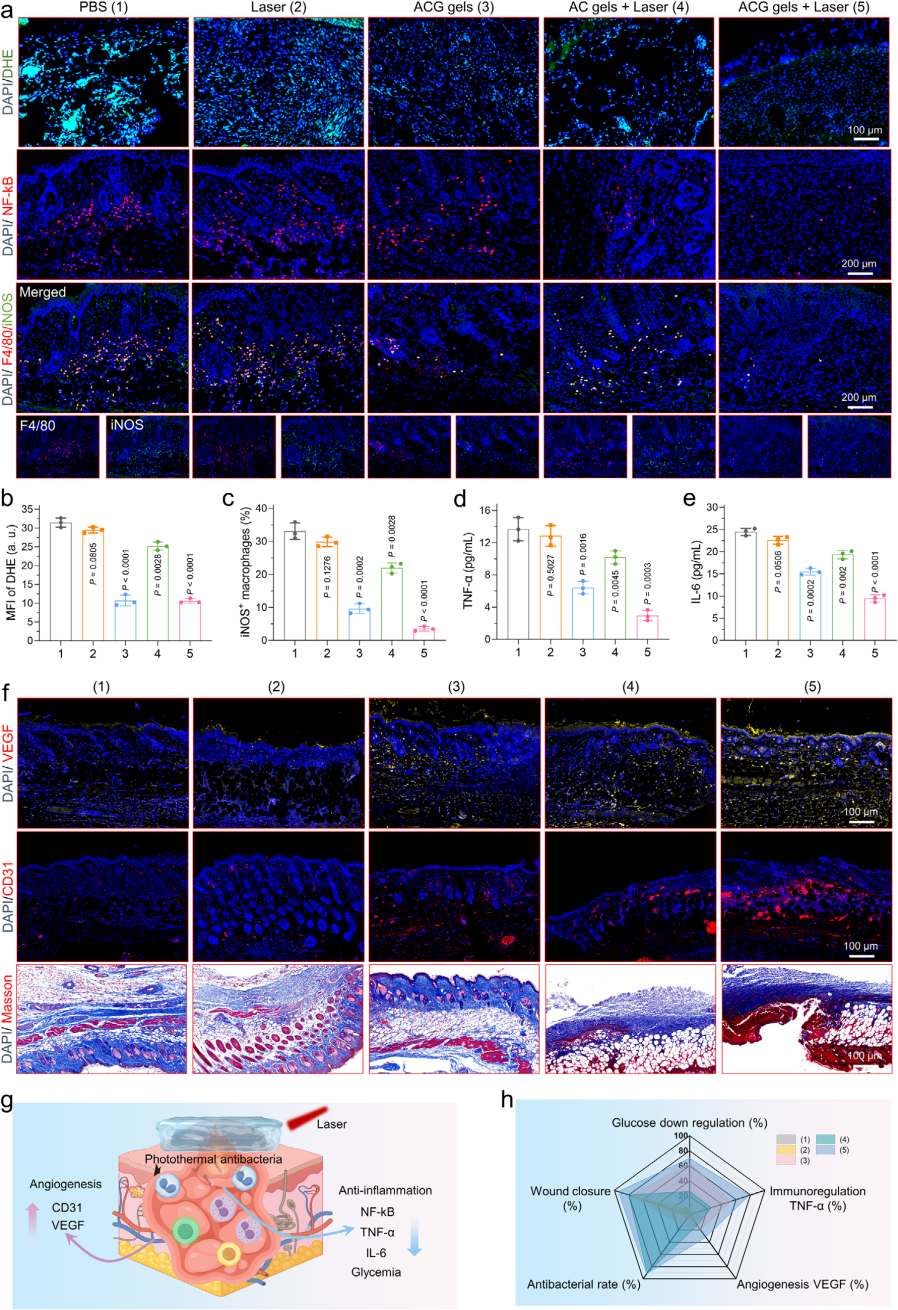
Mechanisms of ACG gel in promoting the healing of diabetic infected wounds. a–c) Representative immunofluorescence images and corresponding semiquantitative data for DHE, NF‐kB, and F4/80/iNOS‐stained sections of skin tissues from mice 3 days post‐treatment with different modalities. Levels of d)TNF‐α and e) IL‐6 produced around the wound in different treatment groups at day 3 after different treatments. f) Representative images of VEGF, CD31 and Masson‐stained sections of skin tissues from mice at day 3 after different treatments. g) Operational mechanisms of ACG gels in infected diabetic wound healing, and h) comprehensive presentation of indicators (immunoregulation, collagen sedimentation, antibacterial effects, angiogenesis, and wound healing) using a radar chart. All data are presented as mean ± SD. Statistical significance was calculated using one‐way ANOVA. ns *p* > 0.05, ^*^ *p* < 0.05, ^**^ *p* < 0.01, ^***^ *p* < 0.001 and ^****^ *p* < 0.0001.

Subsequently, we investigated the mechanism underlying the photothermal antibacterial enhancement of diabetic wound healing in the presence of an infection using ACG gels. Initially, a series of biochemical indicator assays were performed on wounds three days post‐treatment to identify the factors that facilitate wound healing. Initially, DHE, NF‐κB, and F4/80/inducible nitric oxide synthase (iNOS) double staining experiments were performed to confirm the oxidative stress and inflammation state of the wounds. These immunofluorescence images showed that the ROS levels and expression of iNOS and NF‐κB are significantly lower in the ACG gels + Laser group than in other groups, indicating that ACG gels can protect cells from oxidative stress and inflammation (**Figure** **8**a–c; Figure S19, Supporting Information). While AC gel combined with laser treatment effectively eradicated bacteria, it did not substantially reduce the inflammation levels at the wound site, likely due to tissue damage caused by photothermal effects, which may explain the reduced efficacy of photothermal therapy alone during the later stages of treatment. In addition, the inflammatory state of the wounds after the different treatments was evaluated (IL‐6 and TNF‐α are typical pro‐inflammatory factors). Consistent with the immunofluorescence results, wounds in the ACG gel + laser group exhibited a slight inflammatory state compared to the other groups, confirming the anti‐inflammatory effect of ACG gels (Figure 8d,e; Figure S20, Supporting Information). Finally, the formation of new blood vessels and collagen deposition in the wound, which are typical indicators of wound remodeling, were investigated after different treatments. Figure 8f illustrates that group 5 exhibited the highest VEGF signal upregulation, the most newly formed vessels, and the densest collagen fibers, indicating the superior therapeutic effects of ACG gels + laser treatment. In addition, we explored the genetic changes in the wound after ACG‐mediated treatment using RNA sequencing (Figure 21). The analysis of transcriptome (GO and KEEG enrich) uncovered that ACG‐mediated antibacterial and antioxidant therapy inhibited immune‐associated signaling pathway (TNF signaling pathway, Toll‐like receptor signaling pathway, and IL.17 signaling pathway), resulting in the control of the local inflammatory response and thus promotes wound healing. In conclusion, ACG gels demonstrated excellent healing ability toward infectious diabetic wounds owing to their remarkable antibacterial, antioxidant/anti‐inflammatory, and local glycemic control characteristics under laser irradiation (Figure 8g,h).

Furthermore, the in vivo biocompatibility of the ACG gels was evaluated. Body weight data (Figure S22, Supporting Information) indicated an upward trend across all groups, suggesting the safety of all samples in mice. Figure S23 (Supporting Information) illustrates the absence of pathological malformations and organ damage in each group. Additionally, all serum biochemical indicators remained within normal ranges (Figure S24, Supporting Information). These findings suggest that ACG gels exhibit no significant systemic toxicity during the treatment period and hold promise as a reliable platform for wound healing in infectious diabetic wounds.

## 3 Conclusion

In summary, we successfully developed a multifunctional hydrogel dressing (ACG gel) for the treatment of diabetic wounds. This system exhibited capabilities for local glycemic management, as well as antioxidant and photothermal antibacterial activities. ACG gels effectively captured and eliminated ROS, thereby protecting wound cells from oxidative damage in vitro. Additionally, in the hyperglycemic microenvironment of diabetic wounds, ACG gels released Gox, effectively reducing local glucose levels and alleviating oxidative stress. In both in vitro and in vivo experiments, ACG gels exhibited exceptional photothermal conversion properties, enabling effective sterilization through the synergistic effects of energy deprivation and photothermal bacterial ablation. In infected wounds, NIR‐mediated photothermal therapy using ACG gels effectively eradicated bacterial infections mitigated the inflammatory response and oxidative stress associated, and regulated local glycemia. These combined effects promoted angiogenesis and tissue regeneration. Overall, the properties of ACG gel dressings highlight their significant clinical potential for the treatment of diabetic wounds.

## 4 Experimental Section

### Statistical Analysis

All statistical data were presented as means ± standard deviations (SD) and were evaluated using GraphPad Prism 8.5 (GraphPad, USA). All flow cytometry data were analyzed on the FlowJo software package (version 10). Living imaging software (VISQUE In vivo Smart‐LF) was used to analyze bioluminescent and fluorescent images. One‐way analysis of variance (ANOVA) with Tukey's multiple comparisons test was used for multiple‐group comparisons. All experiments were repeated at least three times. The calculated probability (p) was distinguished as (ns *P* > 0.05), (^*^ *P* < 0.05), (^**^ *P* < 0.01), (^***^ *P* < 0.001), and (^****^ *P* < 0.0001).

### Animal Experiments

All animal experiments were under the context of the animal protocols approved by the Institutional Animal Care and Use Committee guidelines in Shanghai Tenth Peoples’ Hospital (protocol number: SHDSYY‐2024‐6461). All mice were kept in accordance with the policies on animal research of the National Ministry of Health. All mice used for animal experiments are female with a gentle character, and the sex of the mice does not affect the results of the experiments.

## Conflict of Interest

The authors declare no conflict of interest.

## Supporting information



Supporting Information

## Data Availability

The data that support the findings of this study are available from the corresponding author upon reasonable request.
