# Supplementary material for: A Tailored Hydrogel With Local Glycemia Management, Antioxidant Activity, and Photothermal Antibacterial Properties for Diabetic Wound Healing
Source: Adv Sci (Weinh). 2025 Mar 5;12(16):2414161. doi: 10.1002/advs.202414161 (PMC12021113; doi:10.1002/advs.202414161)
Supplement: Supplementary file 1 — Supporting Information [file ADVS-12-2414161-s001.docx]

***Supporting Information for:***

**A Tailored Hydrogel with Local Glycemia Management Antioxidant Activity and Photothermal Antibacterial for Promotion of Diabetic Wound Healing**

Bangguo Zhou^2^, Yangying Duan^1^, Wenhao Li^1^, Tao Chen^2^, Jincheng Wang^2^, Manting Cao^3^, Guohao Lin^3^, Ke Yang^1*^, Zhangqi Lai^3*^, Wencheng Wu^1*^

^1^Central Laboratory and Department of Medical Ultrasound, Sichuan Academy of Medical Sciences, Sichuan Provincial People’s Hospital, University of Electronic Science and Technology of China, Chengdu 610072 Sichuan, P. R. China. E-mail: [wuwencheng@uestc.edu.cn](mailto:wuwencheng@uestc.edu.cn).

^2^Department of Radiology, The First Affiliated Hospital, College of Medicine, Zhejiang University, Hangzhou 310003, Zhejiang, P. R. China.

^3^The Third School of Clinical Medicine (School of Rehabilitation Medicine), Zhejiang Chinese Medical University, Hangzhou, P. R. China. E-mail: laizhangqi@126.com.

***Supplementary methods***

**Materials:** Sodium borohydride (NaBH_4_, 99%), hexadecyltrimethylammonium bromide (CTAB, for molecular biology, ≥ 99.0%), silver nitrate (AgNO_3_, ≥ 99.0%), L-ascorbic acid (AA, ≥ 99.0%), cerium (Ⅲ) acetate hydrate (Ce(AC)_3_⋅xH_2_O, 99.9%), Glucose oxidase (GOx), and 9, 10-anthracenediyl-bis (methylene) dimalonic acid (ABDA, ≥ 90%) were purchased from Sigma-Aldrich. Tetrachloroauric (Ⅲ) acid tetrahydrate (HAuCl_4_⋅4H_2_O), hydrochloric acid (HCl, ~36.0−38.0 w%), dimethylformamide (DMF), tetrahydrofuran (THF), N,N,N′,N′-tetramethyl-1,3-propanediamine (1.5 mmol), 4- 3 (bromomethyl) phenylboronic acid, and methanol (CH_3_OH, ≥ 99.5%) were obtained from Sinopharm Chemical Reagent. Cerium nitrate hexahydrate (Ce(NO_3_)_3_⋅6H_2_O, ≥99.5%), potassium sodium tartrate tetrahydrate (C_4_H_4_O_6_KNa⋅4H_2_O, 99%), salicylic acid (C_6_H_4_(OH)COOH, ≥ 99.0%), sodium hypochlorite solution (NaClO, available chlorine ≥ 5.0%) and ammonium chloride (NH_4_Cl, PT) were purchased from Aladdin Reagent. Potassium tetrachloroplatinate (Ⅱ) (K_2_PtCl_4_) and chloroplatinic acid hexahydrate (H_2_PtCl_6_⋅6H_2_O) were obtained from Shenyang Research Institute of Nonferrous Metals. Deionized (DI) water was used in all experiments. RPMI-1640 (Roswell Park Memorial Institute), penicillin/streptomycin solution, and trypsin-ethylene diamine tetra-acetic acid (Trypsin-EDTA, 0.05%) were purchased from Gibco BRL (Gaithersburg, USA). Roswell Park Memorial Institute 1640 (RPMI 1640),3-[4,5-Dimethylthiazol-2-yl]-2, 5-diphenylte-trazolium bromide (MTT), phosphate-buffered saline (PBS), and dimethyl sulfoxide (DMSO) were purchased from Biosharp (Anhui, China). All reagents were used as received without further processing. Calcein, 4,6-diamidino-2-phenylindole (DAPI), propidium iodide (PI), fluorescein isothiocyanate (FITC), Superior Fetal bovine serum (sFBS, U11-020A), and cell counting Kit-8 (CCK-8) were purchased from YOBIBIO (Shanghai, China).

**Fabrication of Au-CeO_2_ dumbbells.** Initially, the synthesis of the starting gold nanorods (Au NRs) was conducted using a modified seed-mediated growth method.1,2 The seed solution was prepared by injecting a freshly prepared, ice-cold NaBH_4_ solution (10 mM, 600 μL) into a mixture of HAuCl_4_ (10 mM, 250 μL) and CTAB (0.1 M, 9.75 mL), followed by rapid inversion for 2 minutes. The resulting seed solution was maintained at room temperature for 2 hours before use. The growth solution was prepared by sequentially adding HAuCl4 (10 mM, 2 mL), silver nitrate (AgNO_3_, 10 mM, 400 μL), and HCl (1 M, 800 μL) to a CTAB solution (0.1 M, 40 mL), followed by the addition of a freshly prepared AA solution (0.1 M, 320 mL). After the solution became colorless, the seed solution (Au NR samples) was added to the growth solution and mixed by inverting for 2 minutes. The mixture was then left undisturbed for at least 6 hours. The as-synthesized Au NRs solution (10 mL) was subjected to centrifugation and subsequently washed with deionized water (30 mL) to eliminate excess surfactant. The Au NRs were then re-dispersed in a CTAB solution (0.1 mM, 5 mL) within a 15-mL centrifuge tube. Following this, a K_2_PtCl_4_) solution (0.1 mM, 200 μL) was introduced into the Au NRs solution with gentle agitation. The resulting mixture was maintained at ambient temperature for 2 minutes to facilitate the adsorption of PtCl_4_^2-^ ions onto the Au NRs. Subsequently, a freshly Ce(AC)_3_ solution (10 mM, 500 μL) and deionized water (4.3 mL) were added sequentially to the Au NRs solution, again under gentle agitation. The total volume of the prepared solution was 10 mL, with a CTAB concentration of 50 μM. This solution was subsequently incubated in an oven at 100 °C for 1 hour to synthesize the Au-CeO_2_ dumbbells. The resulting product was purified through centrifugation and then redispersed in 10 mL of deionized water for subsequent applications.

**Synthesis of TSPBA.** A solution of N,N,N′,N′-tetramethyl-1,3-propanediamine (1.5 mmol) and 4-(bromomethyl)phenylboronic acid (1 g) was prepared in dimethylformamide (DMF) (40 mL) and subjected to magnetic stirring in a water bath for 24 hours at 60°C. Subsequently, the resulting clear solution was introduced into tetrahydrofuran (THF) (100 mL). The resultant white precipitate was isolated and washed thrice with THF (20 mL each). The pure TSPBA was obtained following vacuum drying under low-temperature conditions overnight.

**Synthesis of ACG gels dressing.** Polyvinyl alcohol (PVA, 1 g) was dissolved in 20 mL of deionized water, and the temperature was gradually elevated to 95°C under continuous stirring to achieve a clear solution. Subsequently, a mixture of 5 wt% trisodium phosphate borate (TSPBA) in water (2 mL) and 5 wt% PVA in water (2 mL) was prepared to form a hydrogel dressing. To fabricate Au-CeO_2_ dumbbells and GOx-loaded ACG gels, 5 mg of Au-CeO_2_ dumbbells and 2.50 mg of GOx were dissolved in an aqueous PVA solution. During the in vivo experiments, PVA solutions (with or without incorporated drugs) and TSPBA solutions were initially maintained at room temperature for 30 minutes. Subsequently, these solutions were administered into the tumor using a double-barrel syringe with a diameter of 0.45 mm to facilitate gel formation.

**ROS-scavenging activities of** **Au-CeO_2_ dumbbells and ACG gels.** A Superoxide Dismutase (SOD) Assay Kit was utilized to evaluate the scavenging capacity of Au-CeO_2_ dumbbells and ACG gels for superoxide anions (O_2_^·-^). Initially, a dispersion of CeSACs was introduced into a working solution containing water-soluble tetrazolium salt (WST), which specifically reacts with O_2_^·-^. Subsequently, xanthine oxidase was added to the mixture, which was then thoroughly homogenized, resulting in final Au-CeO_2_ dumbbells and ACG gels containing an equivalent concentration of Au-CeO_2_ at 20 μg/mL. After an incubation period of 20 minutes at 37 °C, the absorbance of the different solution groups was measured at a wavelength of 450 nm. Electron Spin Resonance (ESR) analysis: The O_2_^·-^ generated via the photolysis of riboflavin (50 μL, 6 mM) was utilized as a substrate to assess the scavenging efficacy of different samples. The spin-trap agent 5,5-Dimethyl-1-pyrroline N-oxide (DMPO) was employed to capture the O_2_^·-^, resulting in the formation of the DMPO-O_2_^·-^ adduct, which displayed characteristic peaks in the ESR spectra. The variations in the intensity of these peaks were subsequently analyzed to evaluate the O_2_^·-^-scavenging capacity of Au-CeO_2_ dumbbells and ACG gels.

The ·OH  scavenging activity of Au-CeO_2_ dumbbells and ACG gels was evaluated using a microplate reader by assessing the effective elimination of ·OH generated via the Fenton reaction. A solution comprising FeSO_4_ (1 mM) and H_2_O_2_ (2 mM) in sodium acetate buffer was prepared, to which various samples (Au-CeO_2_ dumbbells and ACG gels, each containing an equivalent concentration of Au-CeO_2_ at 20 μg/mL) were added. Following a reaction period of 5 minutes, 3,3',5,5'-tetramethylbenzidine (TMB) at a concentration of 5 mM was introduced, and the absorbance of the solution was measured at 650 nm. The capacity of the different samples to scavenge hydroxyl radicals produced by the Fenton reaction was further corroborated. ESR spectroscopy using the above method.

Ammonium molybdate (AM) was utilized for the quantification process via colorimetric development following its reaction with H_2_O_2_. The H_2_O_2_ was subjected to Au-CeO_2_ dumbbells and ACG gels, each containing an equivalent concentration of Au-CeO_2_ at 20 μg/mL for 10 minutes, after which AM was added to the system. Absorbance measurements were then conducted at a wavelength of 405 nm. Simultaneously, the oxygen content in the solution was assessed using a dissolved oxygen meter.

**Cell culture**. L929 mouse fibroblast cells (L929 cells) and human umbilical vein endothelial cells (HUVECs) were obtained from the China Center for Type Culture Collection (Wuhan, China). Bone marrow (BM)-derived macrophages (BMDMs) were isolated from the bone marrow of mice. All cells were incubated at 37°C under a humidified atmosphere containing 5% CO_2_, and the medium was refreshed every 2-3 days.

**In vitro ROS-scavenging activities of** **Au-CeO_2_ dumbbells and ACG gels:** To investigate the protective effects against H_2_O_2_-induced oxidative stress, Au-CeO_2_ dumbbells and ACG gels (each at an equivalent concentration of Au-CeO_2_ at 20 μg/mL) were cultured withL929, HUVEC, and BMDMs cells for 24 hours, both in the absence and presence of H_2_O_2_ (200 µM). Cell viability was assessed using the MTT assay. The relative genes and mRNA expression levels of BMDMs after different treatments were determined by RNA sequencing and quantitative reverse transcription polymerase chain reaction (RT-PCR). For confocal imaging and additional flow cytometry assays, L929 and BMDMs were cultured with the aforementioned materials in the absence or presence of H_2_O_2_ (400 µM). After a 2-hour incubation period, 20 µM dichlorofluorescein diacetate (DCFH-DA) was introduced to assess intracellular ROS generation.

**In vitro photothermal effect and** **photothermal antimicrobial activity.** To evaluate the photothermal efficacy of Au-CeO_2_ dumbbells and ACG gels, aliquots of 100 μL, each containing an equivalent concentration of Au-CeO_2_ at 20 μg/mL, were distributed into a 96-well plate. These samples were subjected to irradiation using an 808 nm laser (New Industries Optoelectronics Technology Co., Ltd., Changchun, China) at varying power densities of 0.5, 0.75, 1, 1.25, and 1.5 W/cm² for 5 minutes. Temperature measurements and thermal imaging were conducted at one-minute intervals utilizing a FLIR thermal camera (FORTRIC225, Shanghai Thermal Image Electromechanical Technology Co. Ltd., China).

The Gram-positive bacterium Staphylococcus aureus (*S. aureus*) and the Gram-negative bacterium Escherichia coli (*E. coli*) were cultured in a standard Luria-Bertani (LB) medium devoid of antibiotics and incubated in a shaking incubator set at 37°C and 200 rpm for a duration of 12 to 16 hours. The optical density of the bacterial suspension at 600 nm was measured using a microplate reader (TECAN, Switzerland). The in vitro photothermal antimicrobial efficacy of Au-CeO_2_ dumbbells and ACG gels against *S. aureus* and *E. coli* was quantitatively evaluated employing the plate count method and a live/dead staining assay for bacterial viability. Briefly, Au-CeO_2_ dumbbells and ACG gels were added into *S. aureus* (10^6^ CFU/mL) or *E. coli* (10^6^ CFU/mL) to achieve a final equal Au-CeO_2_ dumbbells concentration of 20 µg/mL. Subsequently, the *S. aureus* and *E. coli* suspensions were divided into five groups: 1) control, 2) Laser, 3) Au-CeO_2_, 4) Au-CeO_2_ + Laser, and 5) ACG + Laser. Before irradiation, the bacteria were incubated with Au-CeO_2_ dumbbells or ACG gels for 6 h at 37°C. Group 2, 4, and 5 bacteria were irradiated with an NIR laser (808 nm, 1.5 W/cm^2^ ) for 5 min. Subsequently, the bacterial suspension was diluted with PBS and 100 µL of the resulting dilution was plated onto LB agar. Following incubation, the CFUs were enumerated. Bacterial viability was also measured by a standard bacteria MMT assay. For the live/dead staining assay, the bacteria were stained with the SYTO9/PI Viability Kit at 37°C for a duration of 30 to 60 minutes. The stained bacteria were then placed onto clear glass slides and visualized using a confocal laser scan microscope. And, the morphology of treated bacteria was obtained by SEM.

**Mouse model of diabetic wounds.** The impact of ACG gels on the healing of diabetic wounds was assessed using a type-1 diabetes mouse model characterized by cutaneous wounds. To establish the streptozotocin (STZ)-induced type-1 diabetes model, female Babl/c mice, aged six weeks, received intraperitoneal injections of STZ at a dosage of 50 mg/kg for five consecutive days. Following two weeks, blood samples were collected via the tail vein, and blood glucose levels were measured using a glucometer. Mice exhibiting blood glucose levels exceeding 15 mM were classified as diabetic and were maintained under standard conditions for an additional four weeks before the creation of full-thickness cutaneous wounds. Subsequently, a full-thickness circular cutaneous wound with a diameter of 20 mm was induced on the dorsal region of the mice. The diabetic mice with wounds were randomly allocated into five experimental groups (n = 5 per group): the control group, which received 150 μL of PBS; the free gels group, treated with 150 μL of free gels; the G gels group, administered 150 μL of G gels hydrogel; the AC gels group, which received 150 μL of AC gels; and the ACG gels group, administered 150 μL of ACG gels. The hydrogel treatments were applied to the wounds and replaced every two days. Photographic documentation of the wounds was conducted on days 0, 3, 5, and 7. The wound areas were quantified and analyzed using ImageJ software. The relative wound size was calculated using the formula: Relative wound size (%) = (Area_n_/Area_0_) × 100%, where Area_0_ represents the initial wound area and Area_n_ denotes the wound area at subsequent time points.

**In vivo photothermal effects.** To evaluate the in vivo photothermal effects of the Au-CeO_2_ dumbbells and ACG gels, a full-thickness round cutaneous wound was induced on the backs of diabetic mice. The wounded mice were randomly divided into five groups: Free gels + Laser (150 µL Gel), AC gels + Laser (150 µL AC gels), and ACG gels + Laser (150 µL ACG gels). The wounds were irradiated with an NIR laser (808 nm, 1.5 W/cm^2^ ) for 5 min after adding hydrogels. Thermal images of the wound area were immediately recorded using a FLIR thermal camera.

**Mouse model of infected wounds.** The impact of ACG gels on the healing of infected wounds was assessed utilizing a murine model of cutaneous infection. Specifically, female BALB/c mice, aged six weeks, were anesthetized, and a full-thickness circular cutaneous wound, measuring 20 mm in diameter, was created on their dorsum following shaving. Subsequently, 50 µL of a *S. aureus* suspension, at a concentration of 2×10^8^ CFU/mL, was applied to each wound site and maintained for a duration of two days. Thereafter, either phosphate-buffered saline (PBS) or hydrogels were administered directly into the *S.aureus*-infected wounds. The mice with infected wounds were randomly allocated into five groups, each comprising five individuals (n=5): (1) PBS, (2) Laser, (3) ACG gels, (4) AC gels + Laser, and (5) ACG gels + Laser. In the NIR groups, the infected wound areas were irradiated with a NIR laser (808 nm, 1.5 W/cm^2^ ) after adding hydrogels for 5 min. Wounds were photographed on days 0, 3, 5, 7, and 9. The methodology for measuring wound area was consistent with that employed for diabetic wounds. To assess the bacterial load in infected wounds post-treatment, a composite sample of the implant and scab was collected from the wound on day 3 and subsequently homogenized. The resulting suspension was serially diluted with PBS and cultured on LB agar plates. Colony-forming units (CFUs) were enumerated to estimate the residual bacterial population.

**Detection of wound ROS levels.** In the diabetic and infected diabetic wound models, tissue samples were collected on the third-day post-injury. Subsequently, these samples were prepared as cryosections and subjected to dihydroethidium (DHE) staining. Fluorescence microscopy was employed to capture images, which were then analyzed utilizing ImageJ software.

**In vivo tests of glucose content** **around the wound in diabetes mice models:** The peripheral blood of the wound was collected from ACG gels-treated diabetic mice at different time points (0, 2, 4, 6, 8, 12, 24, 48, 72 h)and the blood glucose level was monitored with the glucose meter (GA-3, Sinocare Inc, China). The untreated mice were used as control.

**Histological and immunofluorescence analysis.** On the third day, wound tissue samples were collected from both diabetic and infected wound models. These samples were subsequently fixed in 4% paraformaldehyde, embedded in paraffin, and sectioned into 5-μm-thick slices. To assess inflammatory responses, sections from the infected wound samples obtained on day 3 were subjected to immunofluorescent staining for IL-6 and TNF-α using antibodies from Abcam (USA), with the staining process conducted overnight at 4°C. To assess the extent of wound regeneration and collagen deposition, samples collected on day 3 were subjected to analysis using Hematoxylin and Eosin (H&E) staining as well as Masson's trichrome staining. To evaluate wound angiogenesis, immunofluorescence staining for α-SMA and CD31 was performed using wound tissue samples obtained on day 3. The slides were observed and imaged using a fluorescence microscope and analyzed using ImageJ software.

**In vivo biocompatibility.** To assess the in vivo biocompatibility of ACG gels, primary organs (heart, liver, spleen, lung, and kidney) and serum samples were collected from mice across the five previously described groups on day 9. The organs were fixed in 4% paraformaldehyde, embedded in paraffin, and sectioned into 5-μm-thick slices. These sections were subsequently stained with hematoxylin and eosin (H&E) and examined under a light microscope (IX71, Olympus). Additionally, serum biochemical indicators were analyzed.

***Supplementary Figures***

**Figure S1.** The EDS spectra of Au-CeO_2_ dumbbells.

**Figure S2.** The total XPS spectra of Au-CeO_2_ dumbbells.

**Figure S3.** Frequency dependency of the elastic (G′) and viscous (G″) moduli of ACG gels.


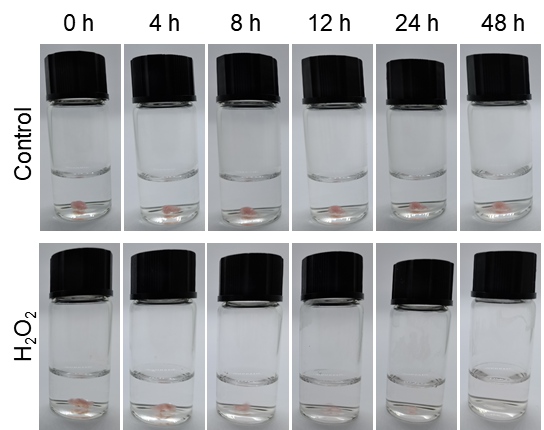


**Figure S4.** Digital photographs of ACG gels immersed in normal PBS and PBS containing H_2_O_2_ (100 μM) at different time points. It is obvious from the figure that after 48 hours, the ACG gels in the H_2_O_2_ group were completely degraded.

**Figure S5.** Changes in glucose content in standard glucose solution with different pH values after the addition of ACG gels. All data are presented as mean ± SD. Statistical significance was calculated by one-way ANOVA. **P* < 0.05, ***P* < 0.01, ****P* < 0.001 and *****P* < 0.0001.


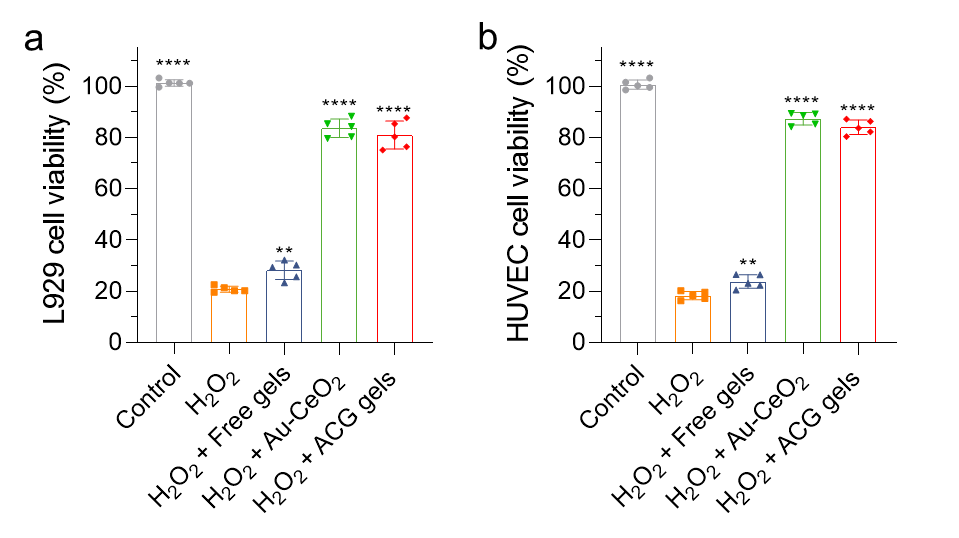


**Figure S6.** The cell viability of (a) L929 cells and (b) HUVEC cells after different treatments. All data are presented as mean ± SD. Statistical significance was calculated by one-way ANOVA. **P* < 0.05, ***P* < 0.01, ****P* < 0.001 and *****P* < 0.0001.


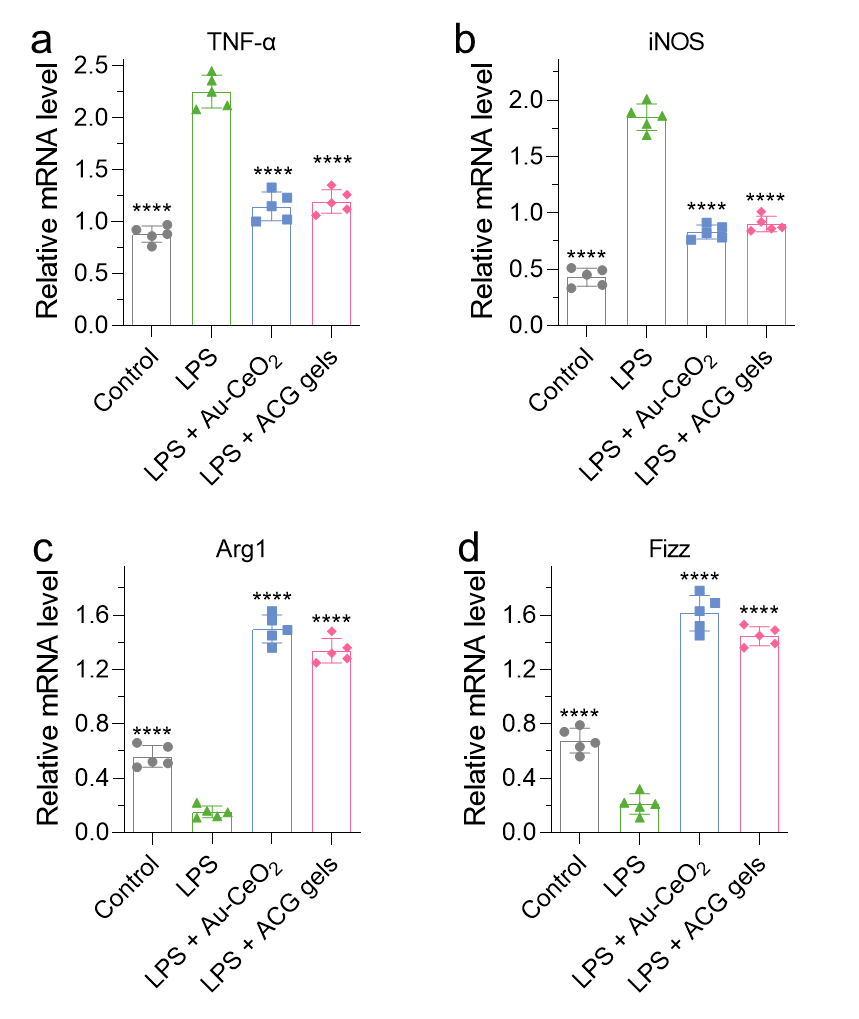


**Figure S7.** Quantitative reverse transcription polymerase chain reaction (RT-PCR) results of (a) TNF-α, (b) iNOS, (c) Arg1, and (d) Fizz mRNA expression in different groups. All data are presented as mean ± SD. Statistical significance was calculated by one-way ANOVA. **P* < 0.05, ***P* < 0.01, ****P* < 0.001 and *****P* < 0.0001.

**Figure S8.** Temperature change of solution containing different photothermal agents after five minutes of irradiation with the same laser power (1W/cm^2^) in the same concentration (200 ppm). All data are presented as mean ± SD. Statistical significance was calculated by one-way ANOVA. **P* < 0.05, ***P* < 0.01, ****P* < 0.001 and *****P* < 0.0001.


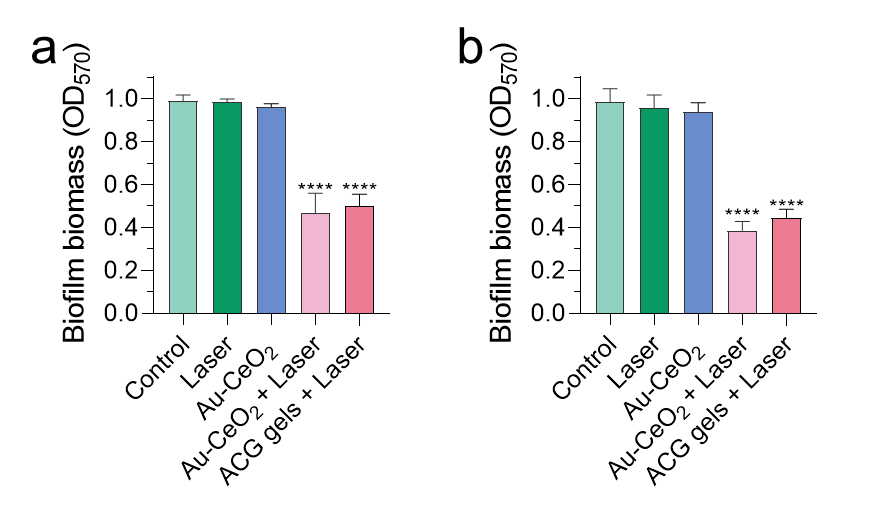


**Figure S9.** Biofilm biomass of *S. aureus* (a) and *E. coli* (b) after different treatments. The units of the Y-axis are arbitrary units (a. u.) representing fluorescence absorption values. All data are presented as mean ± SD. Statistical significance was calculated by one-way ANOVA. **P* < 0.05, ***P* < 0.01, ****P* < 0.001 and *****P* < 0.0001.

**Figure S10.** The cell viability of L929 cells after different treatments. All data are presented as mean ± SD. Statistical significance was calculated by one-way ANOVA. **P* < 0.05, ***P* < 0.01, ****P* < 0.001 and *****P* < 0.0001.

**
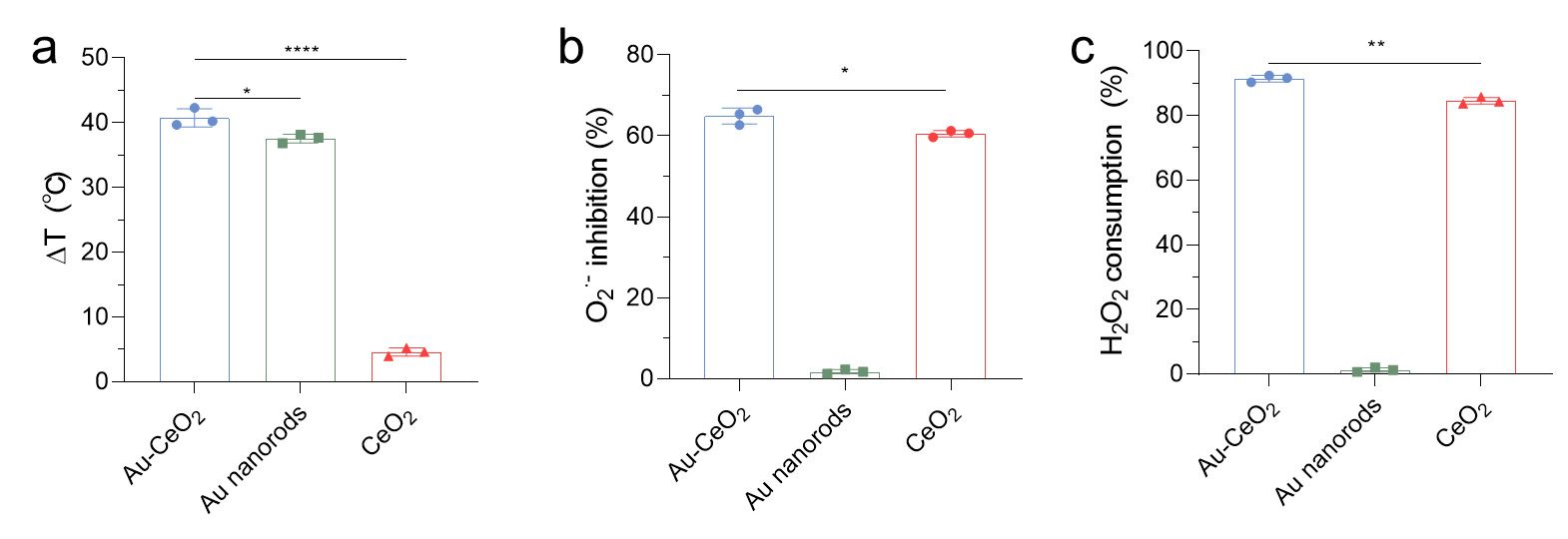
**

**Figure S11.** (a) Temperature change of solution containing different samples after five minutes of irradiation with the same laser power (1W/cm^2^) in the same concentration (200 ppm). (b) The O_2_^·-^ scavenging ability of different samples in the same concentration (200 ppm)was evaluated using an O_2_^·-^-specific total SOD assay kit with WST-8. (c) The H_2_O_2_-scavenging ability of different samples in the same concentration (200 ppm) measured by an H_2_O_2_-specific total CAT assay kit. All data are presented as mean ± SD. Statistical significance was calculated by one-way ANOVA. **P* < 0.05, ***P* < 0.01, ****P* < 0.001 and *****P* < 0.0001.

**
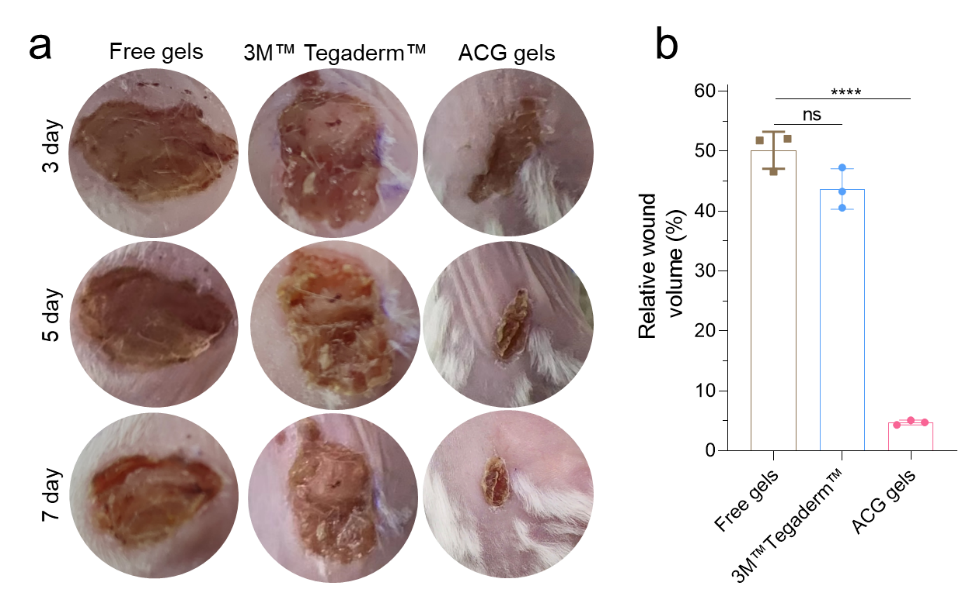
**

**Figure S12.** (a) Representative digital images of diabetic wounds at varied time points after different treatments and corresponding (b) wound healing percentages at the end of treatment in a diabetic wound model. All data are presented as mean ± SD. Statistical significance was calculated by one-way ANOVA. **P* < 0.05, ***P* < 0.01, ****P* < 0.001 and *****P* < 0.0001.

**Figure S13.** Semiquantitative data of fluorescence intensity of DHE in different groups. All data are presented as mean ± SD. Statistical significance was calculated by one-way ANOVA. **P* < 0.05, ***P* < 0.01, ****P* < 0.001 and *****P* < 0.0001.


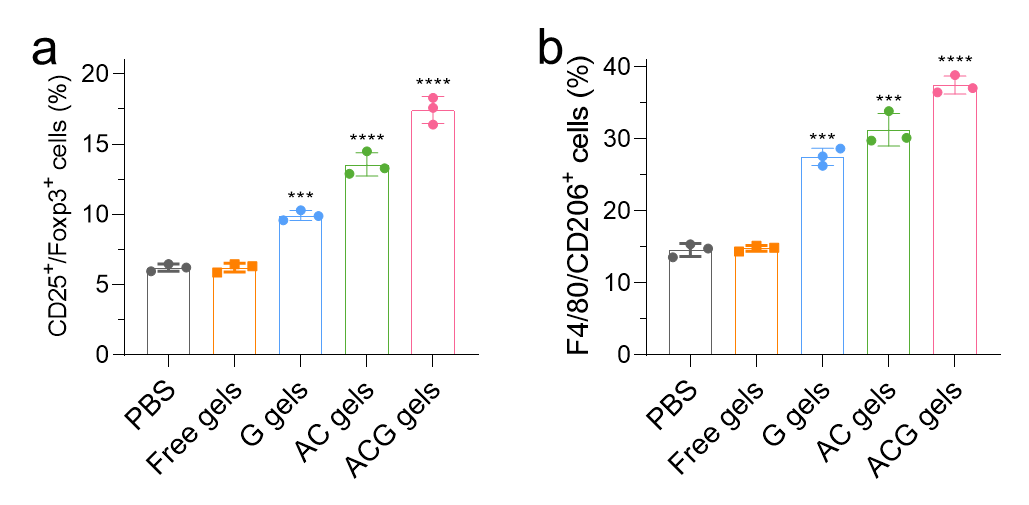


**Figure S14.** The statistic results to determine changes of (a) M2 phenotype macrophages and (b) Tregs in wounds. F4/80^+^CD206^+^ cells were defined as M2 phenotype macrophages. CD4^+^/CD25^+^/Fopx3^+^ cells were defined as Tregs. All data are presented as mean ± SD. Statistical significance was calculated by one-way ANOVA. **P* < 0.05, ***P* < 0.01, ****P* < 0.001 and *****P* < 0.0001.

**Figure S15.** The changing trends in glucose concentration in wounds of mice (n = 3) with and without ACG gel dressings.


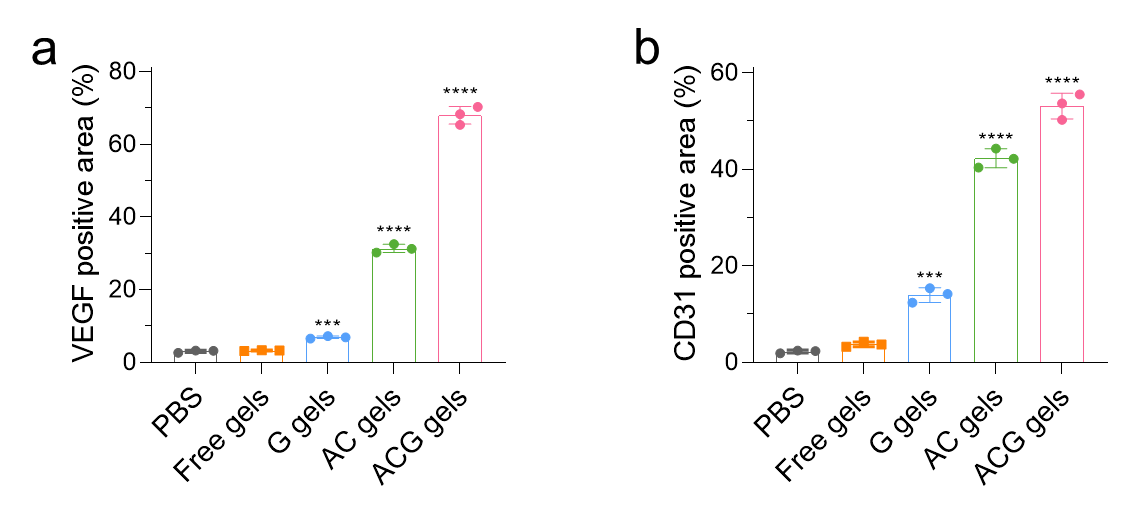


**Figure S16.** Semiquantitative data of fluorescence intensity of (a) VEGF positive area and (b) CD31 positive area in different groups. All data are presented as mean ± SD. Statistical significance was calculated by one-way ANOVA. **P* < 0.05, ***P* < 0.01, ****P* < 0.001 and *****P* < 0.0001.

**Figure S17.** Semiquantitative data of collagen sediment in different groups. All data are presented as mean ± SD. Statistical significance was calculated by one-way ANOVA. **P* < 0.05, ***P* < 0.01, ****P* < 0.001 and *****P* < 0.0001.


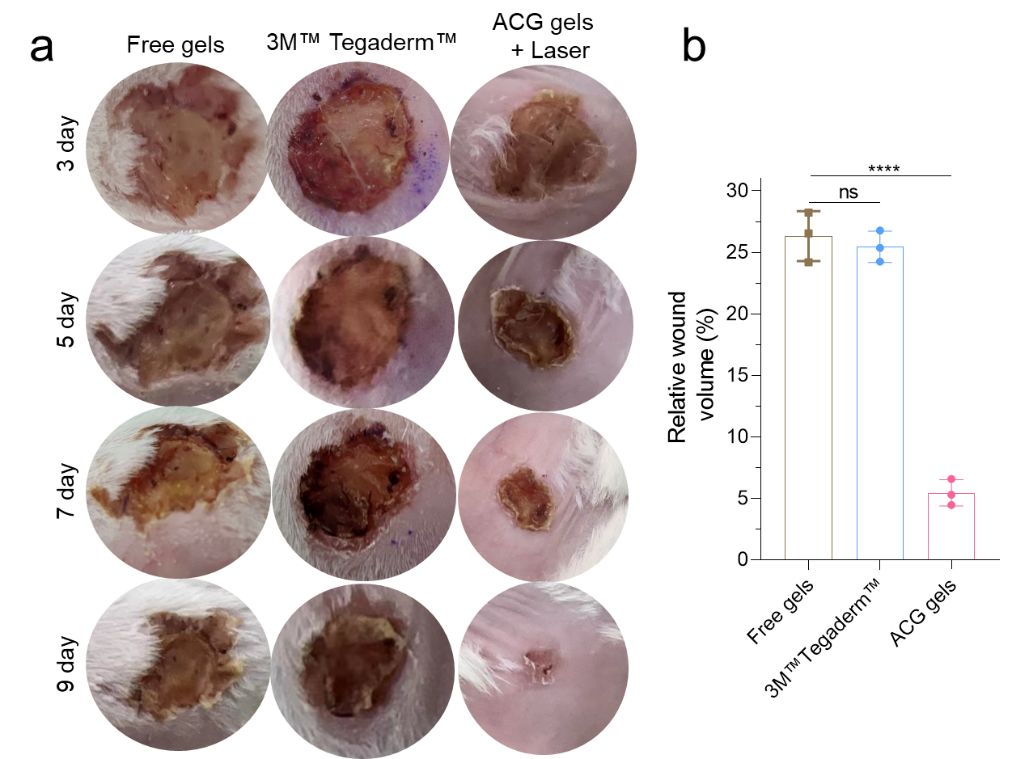


**Figure S18.** (a) Representative digital images of diabetic wounds at varied time points after different treatments and corresponding (b) wound healing percentages at the end of treatment in an infected diabetic wound model. All data are presented as mean ± SD. Statistical significance was calculated by one-way ANOVA. **P* < 0.05, ***P* < 0.01, ****P* < 0.001 and *****P* < 0.0001.

**Figure S19.** Semiquantitative data of NF-kB positive cells in different groups. All data are presented as mean ± SD. Statistical significance was calculated by one-way ANOVA. **P* < 0.05, ***P* < 0.01, ****P* < 0.001 and *****P* < 0.0001.


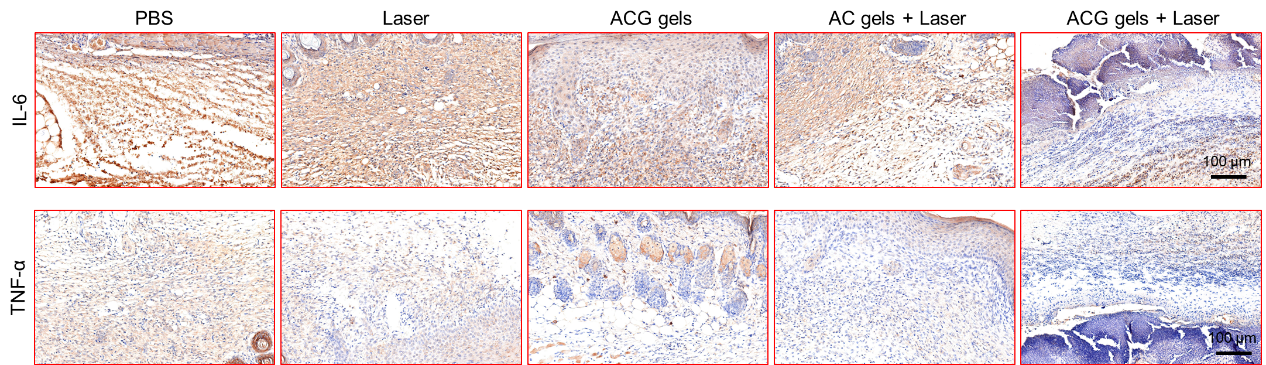


**Figure S20.** IHC staining images of IL-6 and TNF-αafter diverse treatments on day 3.


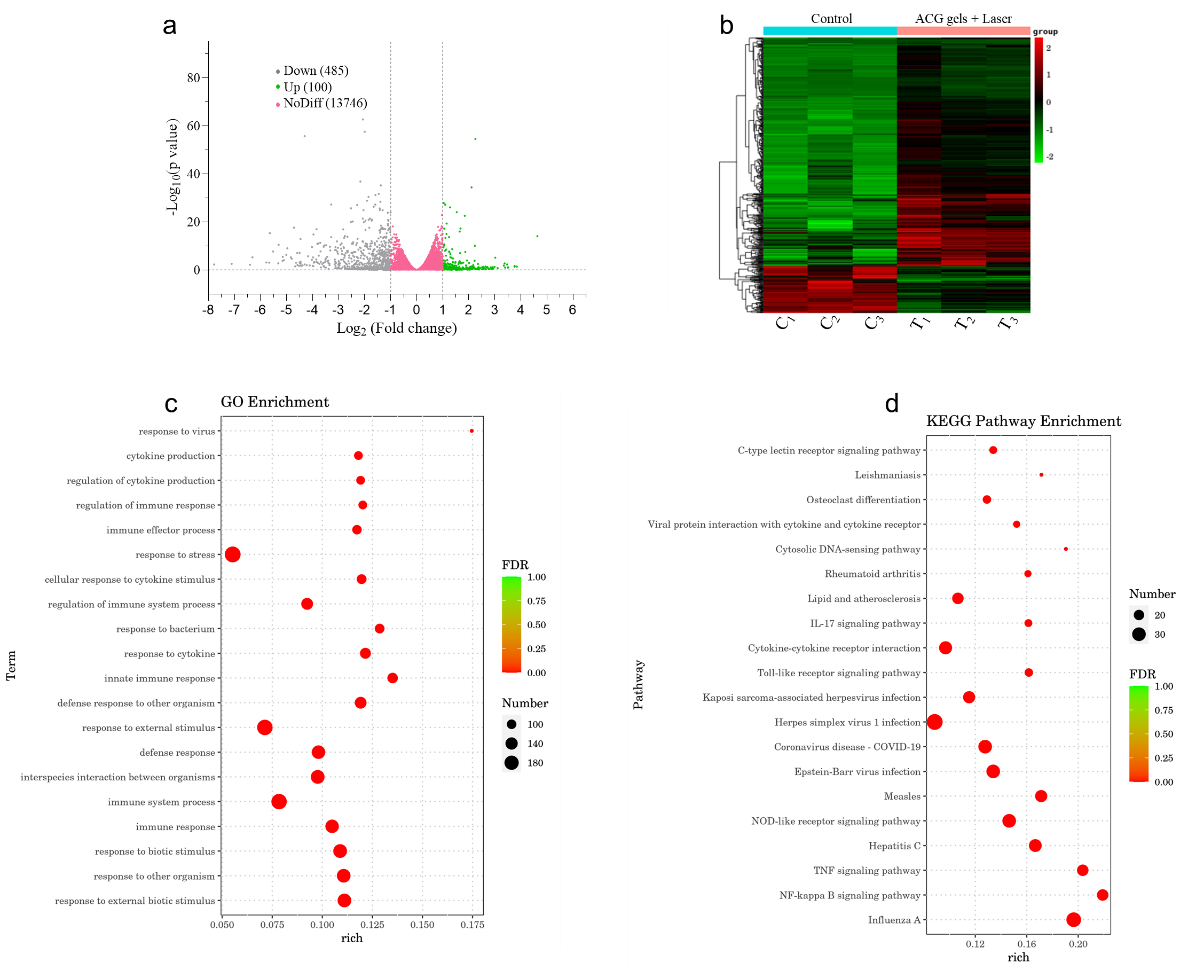


**Figure S21.** (a) Volcano map and (b) heat map of genes alteration with or without ACG gels + Laser treatment (P < 0.05, |fold change | ≥ 2). RNAseq-based c) GO and d) KEGG analysis of differential gene expression profiles after ACG gels + Laser treatment (n = 3 mice per group).

**Figure S22.** Body weight change curves of mice in each group (n = 3) during the treatment period.


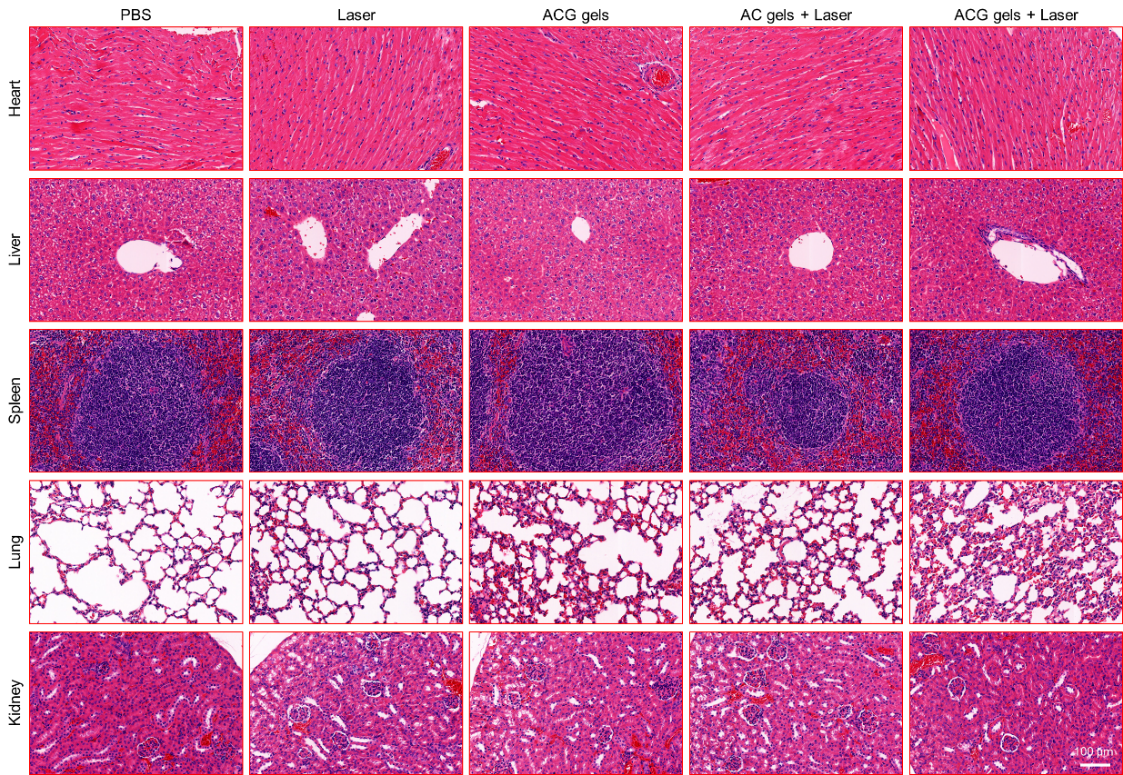


**Figure S23.** H&E staining of the main organs of mice in different groups at the end of treatment.


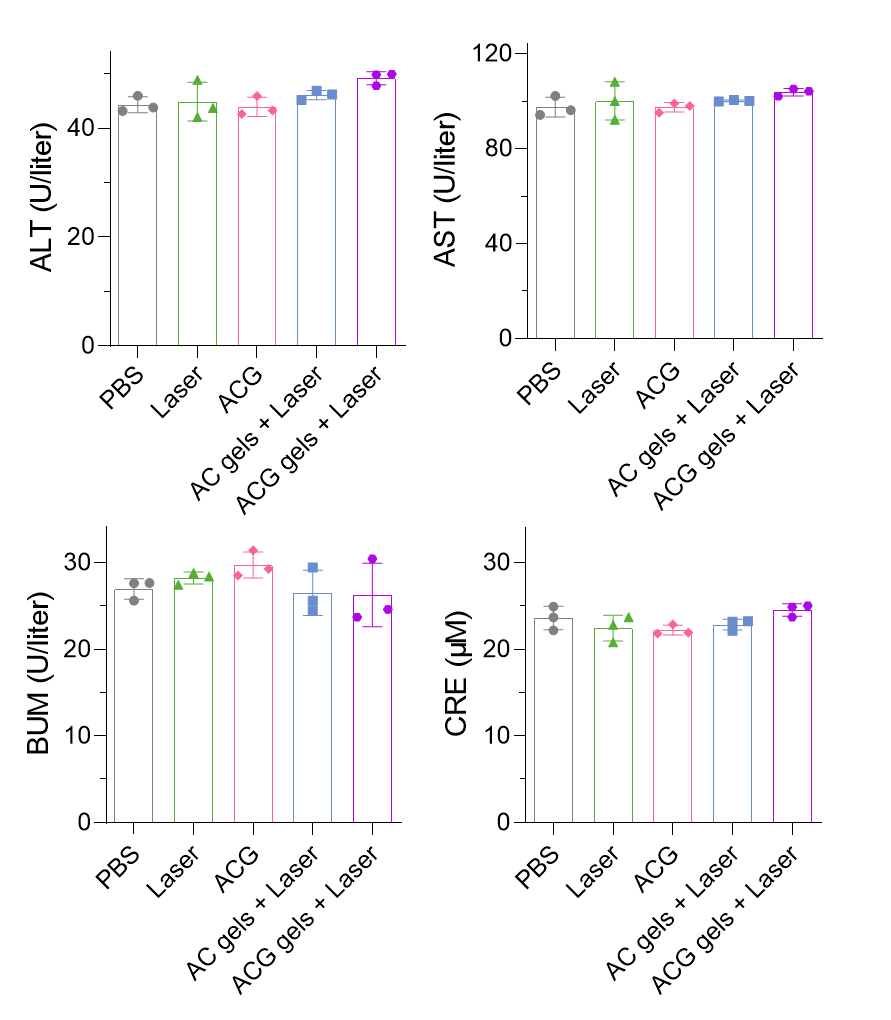


**Figure S24.** The serum biochemistry indicators of mice in different groups at the end of treatment. All data are presented as mean ± SD. Statistical significance was calculated by one-way ANOVA.
